# Consequences of rare diagnoses for education and daily life: development of an observation instrument

**DOI:** 10.1186/s13023-022-02303-y

**Published:** 2022-04-12

**Authors:** Gunilla Jaeger, AnnCatrin Röjvik, Erland Hjelmquist, André Hansla, Kerstin W. Falkman

**Affiliations:** 1 Ågrenska, Gothenburg, Sweden; 2 Department of Psychology, University of Gothenburg, Gothenburg, Sweden

**Keywords:** Educational consequences, Educational methods, Learning, Observation instrument, Rare health conditions, Rare diagnoses

## Abstract

**Background:**

Ågrenska, a Swedish national centre for rare diagnoses and health conditions, has arranged courses for families of children with rare diagnoses for over thirty years, and has experienced that the conditions often have complex and varying consequences in the children´s everyday lives, not least in educational contexts. Knowledge of these consequences and of how to adapt the environment and educational methods is often lacking and the children´s educational needs are not met. Many professionals also report a lack of sources of knowledge. Knowledge formation and dissemination about educational consequences of rare diagnoses are thus of utmost importance. For this purpose, a broad observation instrument was constructed in order to gather knowledge on a group level concerning how functional impairments affect school and everyday situations, how consequences vary within each diagnosis and if there are diagnosis-specific features that lead to specific every day and pedagogical consequences.

**Results:**

The instrument consists of 119 quantitative and 65 qualitative items and covers ten domains: social and communicative ability, emotions and behaviours, communication and language, ability to manage his/her disability and everyday life, activities of daily life, gross and fine motor skills, perception and worldview, prerequisites for learning and basic school abilities. The instrument is intended for use by educational professionals with knowledge of typical development and was content validated against existing instruments. The items were considered relevant as they, with few exceptions, appear in well-known assessment tools. Interrater reliability was based on the observations of six children. Each child was observed by two educators. Interrater reliability was calculated for the quantitative items and items with fixed response options, including yes/no answers, a total of 100 items, which are usually observed during the course. Interrater reliability reached 91%. Factor analysis and Cronbach´s alpha indicated good statistical properties and a multinomial regression gave reasonable results.

**Conclusions:**

The instrument can be used to gather knowledge on a group level of educational and everyday consequences of rare diagnoses. This knowledge can be used to adapt methods and environment to meet the educational needs and create conditions for optimal learning and participation for children with rare health conditions.

**Supplementary Information:**

The online version contains supplementary material available at 10.1186/s13023-022-02303-y.

## Background

In Sweden the term rare health condition is defined as a disease or injury that is found in a maximum of five hundred individuals per million inhabitants. The condition usually leads to persisting functional impairments and consequences for living conditions with special problems due to the rarity [1]. The term includes both diagnosed and undiagnosed rare conditions. The instrument presented in this paper is intended for use with children and adolescents, between the age of 4 and 17, with rare diagnoses.

Although each diagnosis in itself is rare, the total number of children and adolescents with rare diagnoses together constitutes 0.7–0.8 percent of all children and adolescents with life-long disabilities [2], and the number of individuals with rare diagnoses is increasing. One reason is the development of increasingly refined diagnostic methods and treatment.

At the same time, this development leads to preventive measures and early diagnosis reducing the incidence of certain functional impairments. Some diagnoses, which were previously more common, can thereby become increasingly rare over time. Experiences of these diagnoses will then decrease among professionals, implying a risk for a weakening of the collective knowledge. In turn, this creates a demand for sources of structured and easily available knowledge.

As the number of rare diagnoses increases, more and more specific needs will appear and the need for knowledge will increase accordingly. Common to the groups in question is the need for an interdisciplinary approach [3] focusing, not least, on cognitive and behavioural difficulties [4].

Rare health conditions often lead to consequences in everyday life, and there is insufficient knowledge regarding these consequences. The national association Rare Diseases Sweden claims that their members are often met with ignorance by professionals [5], and in a national survey of parents of children with rare diagnoses, more than half testify that school staff have poor knowledge of their child's diagnosis [6].

An English study shows [7] that professionals, including teachers, working with children with one rare and two more common neurodevelopmental conditions (Williams syndrome, Down syndrome and autism spectrum disorders) recognize only the most general and common symptoms and consequences typical for the condition. They are less likely to know about some of the less common symptoms and difficulties. The authors conclude that the general knowledge of professionals might hinder a broad understanding of the child’s needs and difficulties [7].

A similar view is illustrated in an English study [8], in which 204 teachers of children with one of the four rare diagnoses; fragile X, Prader–Willi, Williams and velo-cardio-facial syndrome (also named 22q11 deletion syndrome) participated in a survey. About two thirds of the participating teachers reported limited knowledge of the syndromes, but also that the educational needs of the children with the syndromes did not differ from those of other children with intellectual disability [8], i.e. the diagnosis and the causes of the needs were not regarded as adding important information for the teachers´ work.

It is not uncommon for the medical perspective to be seen as both irrelevant, and sometimes even incompatible, with the special education perspective. It is also not uncommon to work entirely based on the perceived problems, not considering the need for a diagnosis [9]. This can lead to incorrect conclusions as the same consequence, e.g. difficulties concentrating, can have many different causes, and therefore the adjustments will have to be different for different individuals. For example, when a child with Prader Willi syndrome shows difficulties concentrating, e.g. while trying to solve a mathematical task, it is easy to believe that the task is too difficult in relation to the child's cognitive ability, and the teacher will thus give the child an easier task. Difficulties concentrating, however, can also be due to fatigue caused by the diagnosis-specific muscle weakness, which causes the child to put a lot of energy simply into staying upright in a chair. The appropriate adaptation would then be a comfortable chair of the right size with high back support and armrests [10].

In order to adapt the educational setting and working methods to create optimal conditions for learning and participation for students, professionals need to have knowledge about the diagnosis and the symptoms, as well as their consequences for each individual in different situations. The individual´s functioning and needs, the teacher´s competence and methods, the premises and activities, are of equal importance, all mutually affecting each other and the educational outcome.

This is also in accordance with the bio-psycho-social ICF model [11], which stresses the dynamic interaction of biological, personal and environmental factors, all of which are given equal importance, affecting the outcome of an individual’s functioning.

The experience of Ågrenska’s staff is that the professionals on whom the individuals with rare health conditions are dependent often have an insufficient knowledge of the complex, complicated and varying consequences of rare health conditions.

At the same time, Ågrenska has had a unique opportunity to develop knowledge about rare diagnoses, not least by arranging courses for families of children with rare diagnoses for over 30 years. This has provided the opportunity to meet up to ten children with the same rare diagnosis at a time and observe how their health conditions have consequences for learning and everyday life. Since these consequences often are very complex, education and special education for persons with rare health conditions should be characterized by an interdisciplinary approach.

In the Nordic context there is a tradition of offering educational and medical services to everyone, including people with rare health conditions, but despite this there is very little systematic documentation about all relevant specific educational and everyday needs for children and adolescents with rare health conditions.

In the Swedish context the National Board of Health and Welfare's knowledge database [12] on rare health conditions provides high quality information for a broad target group. The focus is primarily on medical and medically related aspects, treatment and support. It is not intended to replace the specialist knowledge of different professionals.

In Norway, there are two national competence centres. Frambu, an interdisciplinary centre arranging activities for individuals with rare health conditions [13] and The Training and Counselling Center, TRS (Trenings- og rådgivningssenteret) at Sunnaas Hospital outside Oslo [14], working with competence development, research and dissemination of information on skeletal and connective tissue diseases, spinal cord hernia and dysmelia [15].

Frambu publishes brochures containing descriptions of diagnoses, and information about available support systems and legal rights for individuals with disability. They also illustrate general consequences of rare diagnoses, diagnosis-specific descriptions of problem areas and needs, as well as suggested interventions and adaptations for preschool and school settings [16].

TRS publishes diagnosis specific information on their website, focusing on medical and diagnostic aspects, treatment and physical adaptions for everyday life. For some diagnoses there are also specific descriptions about cognitive aspects, learning and suggestions for adaptions in school environment and teaching methods [15].

The National Board of Health and Welfare in Denmark has published diagnosis-specific writings on a large number of rare health conditions. The information is primarily related to medical and physical aspects, but also contains some general descriptions of psychomotor development, learning, speech and language as well as suggestions for adaptions [17].

There are published studies on various aspects of rare diagnoses but with little focus on specific educational and everyday needs.

In Sweden there is published research on e.g. Rett syndrome [18–20], Alström syndrome [21, 22] and 22q11 deletion syndrome [23–27]. There are also studies on Prader Willi syndrome [28–30] and girls with Turner syndrome [31].

Both Frambu and TRS also conduct research, publishing studies on various aspects of rare diagnoses [32–34].

To the best of our knowledge, only Frambu and TRS has published extensive, diagnosis-specific information of which some include specific educational aspects, about certain diagnoses or syndromes [15, 16]. Other writings are primarily either overviews or, in some cases, more general descriptions of rare diagnoses or deal with only a few of the symptoms found within a syndrome.

The professionals with whom Ågrenska are in contact often point to a lack of sources of knowledge and support. Because the diagnoses are so rare, there is often no basis for developing knowledge at the local level, e.g. in schools and habilitation clinics. Most rare health conditions are syndromes, i.e. varying combinations of different symptoms to be managed simultaneously. Varying combinations and severity of symptoms also result in variations of consequences within each diagnosis [35]. The consequences can also change over time.

That children with rare diseases face particular problems in school and that meeting their needs poses a challenge for the school system is also highlighted in European research [3, 36]. More research is, however, needed in the field of rare disease disability, not least concerning the need for adapted educational methods [37–39]. It is necessary to increase and spread knowledge of the rare diseases and their implications for the children in the schooling environment [36]. Effective inclusion is dependent on the availability of staff and resources, but also on the awareness and experience concerning rare diseases among the staff involved in health and education interventions for children with rare diseases [40].

It is therefore of the outmost importance to gather and disseminate knowledge about the educational and everyday consequences of rare health conditions.

When reviewing a number of existing instruments for information gathering, nothing was found that focused on abilities, while still identifying problem areas and covering relevant aspects for education and everyday life. Therefore, the need for a new observational instrument was identified.

This paper presents an observation instrument for children and adolescents with rare diagnoses developed at Ågrenska, a Swedish centre for rare diagnoses and health conditions. Many existing instruments are used for diagnostic purposes and therefore focus on identifying difficulties and limitations of functioning. We believe it is important to have, and constantly maintain, an approach that is based as far as possible on abilities and developmental possibilities, while still identifying problem areas.

The focus of the instrument presented here is on observations of functioning relevant for education, school settings and daily life.

Specifically the constructed observation instrument should be able to answer the following questions: (1) How does the functional impairment affect the children’s and adolescents’ school and everyday situation? (2) How do consequences vary within each diagnosis? (3) Is it possible to identify diagnosis-specific features that lead to specific pedagogical and every day consequences?

## Materials and methods

During the process of developing the observation instrument, two of the authors (a special education teacher and a psychologist) had the support of an external inter-professional reference group consisting of a speech therapist, a dentist, an occupational therapist, a physiotherapist and a nurse. All with many years’ experience working with individuals with disabilities.

The group had several meetings. Relevant domains of observation were discussed and suggestions of items put forward. The contents were presented to the Ågrenska inter-professional children´s team, working with the children in the family courses for rare diseases, i.e. an internal expert group. Feedback was reported back to the external expert group. Also the scaling system was discussed. A visual analogue scale was tried and then a scale with four fixed steps.

Domains of observation were chosen with focus on educational aspects that were judged to have an impact on the children’s daily life, as well as areas in which children participating in Ågrenska´s programs have shown difficulties.

### Data collection

The context of the application of the instrument is the family courses.

Approximately 22 family courses targeting different rare diagnoses are arranged every year at Ågrenska. Each course gathers up to 10 or 12 families at a time, all having a child, up to the age of 17, with the same rare diagnosis. Families come from all over Sweden and the whole family attends. The course duration is five days, Monday to Friday. The families stay on the Ågrenska premises.

There are three parallel programs every week, one for parents, one for siblings and one for the child with the rare diagnosis.

Parents take part in lectures and discussions with experts on their child´s diagnosis concerning medical, psychological and educational aspects. They are also given information about legislation and legal rights and available support.

Siblings and the children with the diagnosis take part in our school, preschool and leisure activities, and are also are given information about the diagnosis, adapted to their age and developmental level.

The children´s program is planned, adapted and carried out by our inter-professional children´s team, e.g. teachers, preschool teachers, special education teachers, nurses, social worker and recreational specialist. The educational setting and working methods are adapted to the children´s diagnosis, age and individual needs. The team receives information about individual needs from parents and home schools before the course.

In order for a family to attend a course a formal referral from their home county council is needed and families then apply themselves. There is no fee, all costs are covered by county councils and the state. Loss of income for parents is covered by the Swedish social security system. Most often all families applying for a course can be admitted. Each family only attends once.

Observations were carried out during the adapted preschool, school and leisure activities, which are part of the stay and always within the framework of the teachers' and special education teachers' ordinary work with the children. When planning the school, preschool and leisure schedule and activities, the fact that different abilities should be possible to observe is always taken into account. All observers, i.e. teachers and special education teachers, are familiar with the instrument, its domains and items and know when and during which activities the different abilities should be observed. The ratings are both quantitative and qualitative (yes/no answers and free response/text answers) and always made in relation to what a typically developing child of the same chronological age is expected to accomplish. The observations were carried out during both indoor and outdoor activities. All domains were observed every day in order to obtain a picture as complete as possible. During each of the five days the observers took preliminary handwritten notes either directly in the rating form or on separate paper. Towards the end of the course the observers also discussed with and got additional information from other colleagues working with the children, e.g. nurses, recreational specialists, occupational therapist before noting their final observations and ratings in the form, i.e. the summary ratings were always done after the course.

Some school-related abilities were difficult to observe during the five-day family course. This information was instead obtained from the children’s home schools through a telephone interview in conjunction with sharing experiences related to the current diagnosis with the child’s regular teacher.

Diagnoses for observation were included based on the following criteria; that they are non-progressive, with a general development age above 4 years and about which have been noted a great demand for knowledge through telephone calls and email questions. Children with the chosen diagnoses between the age of 4 and 17, for whom parents had given their consent were observed. When possible depending on developmental level consent was also given by the child.

The first data collection was carried out from 2000 until and including 2006 and the second data collection from 2008 until and including 2017. For the final instrument, see Additional file 1.

The final version of the observation instrument was based on statistical analyses described below, but first we mention the differences between the first and second data collection in terms of terminology and items included (for a full description see Additional file 2). At the start of the second data collection period the terminology used in collection of background information was updated. The concept of *individual integration* in elementary and upper secondary school was abolished and replaced by *with the curriculum of special school* in elementary and upper secondary school. In the original instrument any additional functional impairments, i.e. visual or hearing impairment, epilepsy, intellectual disability, behavioural problems, respiratory problems or heart failure, were rated on a scale. This information was provided by parents, and it was therefore difficult to convey a uniform definition of different levels of impairment. Because of this, as of 2008, the presence of any additional impairment was only indicated with a yes or no answer.

From the start the observation instrument was used for most types of rare diagnoses. However, it was found that in rare health conditions that entail extensive multifunctional impairment and a mental age below four years, all items were rated as “severe problems”. Therefore, the instrument was not used for these conditions after the first data collection period. Two items: *head control* and *can reach for objects* were removed as problems in these areas were always rated as “none”.

In addition to the above, a few further changes were made to the instrument for the second data collection period. Under the heading *Ability to manage his/her disability and everyday life*, there were four items that the observers considered very difficult, or impossible, to rate: *has a positive outlook on life*, *has a sense of coherence*, *has control over his/her life*, and *seems to accept his/her disability*. These were removed. A new item was added: *shows a positive attitude towards his/her environment*. The domain *Social ability* was renamed *Social and communicative ability*. Under the heading *Communication and language*, four items were added: *shows interest in communication*, *shows communicative and linguistic ability only with certain persons and only in certain situations*, and *shows difficulties in finding the right words and expressing him/herself*. These four items were added as these problems had been observed in several rare conditions during the first data collection period.

In 2017, the database for the observations was revised and updated with respect to technical aspects of its structure and function. In connection with this, the instrument was reviewed once again and a few more changes were made. The terminology was also updated; *Impairment* was changed to *disability* and *mental retardation* was changed to *intellectual disability*. The background information regarding the child’s school situation was supplemented with the following alternatives: *preschool class, home schooling* and *hospital school.* The rating of muscle tone as being high, low or alternating was changed to *typical* or *atypical muscle tone*. *Involuntary* movement was changed to *atypical* movement. In earlier versions of the instrument, the expression used was that the child *had* certain abilities. This is now instead expressed as the child *showing* these abilities, e.g., attention, concentration, motivation, self-confidence and empathy. In regard to reading and writing ability, the item *letters and words are reversed*, was removed, as this has never been observed. It was replaced with the items *recognizes all sounds* and *reverses sounds*, items which have often proved relevant. Finally, the last item *interests or especially good skills/knowledge in any areas in relation to age* was removed. This item was often not filled out and when filled out the information usually also appeared elsewhere in the instrument.

The first statistical analyses, based on the instrument version from 2017, resulted in the removal of a total of fifteen items under three different headings. Four items were removed from the domain *Emotions and behaviours: stereotypies, tics, compulsions, self-destructive behaviours* due to low reliability. One item was removed from the domain *Fine motor skills: trembles/is shaky* because it essentially had no response variation (98% no answers). From the domain *Prerequisites for learning* one full sub-domain, *conceptual formation/perception,* consisting of ten items, was removed.

These changes resulted in the final version of the observation instrument described below.

## Results

### Domains and items of observation

A final version of the observation instrument, covering ten domains, was developed (see Additional file 1). The focus of the observations is the educational and everyday consequences of specific rare diagnoses and the instrument is intended for use by educational professionals. The two most extensive domains are therefore school-related, i.e. *Prerequisites for learning* and *Basic school skills*, and divided into three and four sub-domains respectively.

1. *Social and communicative ability* (15 items). E.g., contact with peers and adults, playing and socializing with others and the need for adult support in social situations.
2. *Emotions and behaviours* (14 items): E.g., expressions of anxiety or dissatisfaction, lack of social boundaries, restlessness, lack of impulse control, expressions of self-esteem and empathy.
3. *Communication and language* (14 items). E.g., pronunciation, need for alternative and augmentative communication and technical aids, interest in communication.
4. *Ability to manage his / her disability and everyday life* (11 items). E.g., expressions of well-being, attitude to everyday problems, knowledge of own disability.
5. *Activities of daily life, ADL* (10 items). E.g., food situations, dressing and hygiene.
6. *Gross motor skills* (16 items). E.g., bodily control, flexibility, ability to climb stairs, ability to walk on uneven surfaces, running and jumping.
7. *Fine motor skills* (8 items). E.g., ability to cut, snap buttons, handle small objects, hand preference.
8. *Perception and worldview* (21 items). E.g., body perception, eye-hand and eye-foot coordination, distance assessment, perception of time.
9. *Prerequisites for learning* (29 items). Divided into three sub-domains, a) *gatherings/group activities*, b) *individual work*, c) *ability to assimilate information*.
10. *Basic school skills* (46 items). Divided into four sub-domains, a) *reading*, b) *writing*, c) *mathematics*, d) *physical education*.

The final instrument contains 184 items and information regarding these items is collected from two different sources at two different points in time, and is both quantitative and qualitative. The final observation instrument contains 119 quantitative items assessed using an itemized rating scale and 65 qualitative items.

Included in the 184 items there are twenty-three quantitative and twenty-three qualitative items with free text responses, which relate to abilities that are difficult to observe during the five days of the family course. This information is instead collected after the stay during a telephone interview with the home school in connection with the child’s teacher being informed about Ågrenska's general knowledge and experience of the diagnosis in question. In order for the educational activities during the stay at Ågrenska to be arranged as well as possible, the observations are also supplemented with written information collected before the stay. This information relates to the child's prerequisites for learning, e.g. what works well, what difficulties does the child present, and what adjustments are usually made. This information also supplements the observations.

### Ratings of abilities during observation

The ratings in the observation instrument are both quantitative and qualitative and always made in relation to what a typically developing child of the same chronological age is expected to accomplish.

Difficulties expressed by the children are rated as none (coded 1), mild (coded 2), moderate (coded 3) or severe (coded 4). Mild difficulties means that there are some difficulties, in relation to no difficulties at all. Moderate difficulties affect everyday life most of the time. Severe difficulties entail everything from major problems in everyday life to total inability.

The estimated levels of difficulty were found to be consistent with those estimated using the classification in ICF, with the exception of the level severe difficulty. In the currently described observation instrument this level includes both severe and complete disability, while these are divided into two different levels in the ICF.

For each domain there are also a number of open questions for a qualitative description as a complement to the quantitative estimate.

### Content validity

The final version of the observation instrument as a whole was validated against a number of existing and well-known instruments and assessment tools, e.g. Griffiths, Vineland, PEDI, Conners, SNAP IV, as well as previous literature in the area to ensure that relevant domains of observation had been selected and that the included items were clustered in a relevant way. In this way, content validity was secured. For details regarding each assessment tool, see Additional file 3.

The items included in the developed observation instrument were considered relevant for the stated purpose as they, with only a few exceptions, appear also in other well-known assessment tools, see Additional file 3. What sometimes differs is in which domain or under which heading items are found. These differences are not significant, however, and easily explained. The few items that are not supported by the assessment tools listed in Additional file 3 have nevertheless been retained, as they have been observed on numerous occasions in children with rare diagnoses during Ågrenska's family courses.

Furthermore, following the observation that “All indices of validity have implications for content validity” [41, p. 245], we found that the construct validity of our instrument, as indicated by the quantitative analyses, see below, was very good. “Item analysis, internal consistency indices, and the obtained factor structure also provide essential information about the degree to which an item taps the intended constructs and facets (Smith & McCarthy, 1995)” [41, p. 245]. Though of course construct validity does not guarantee content validity, the positive outcome of the statistical analyses corroborates the sample of scales included in the observation instrument.

### Statistical analyses

The final analyses were based on observations of 267 individuals. Missing values in some of the domains, however, implies 266 observations for the domains *Communication and Language*, *Gross motor skills* and *individual work*, 265 observations for *ADL*, and 243 observations for *Ability to manage his/her disability*. The observations include nine different diagnoses and were carried out from 2000 until and including 2005 and from 2008 until and including 2017. For descriptive statistics for each of the eleven observed domains/subdomains subdivided by diagnosis and gender, see Additional file 4.

The final analyses were performed and presented for the final numbers of items for eleven domains/subdomains (domain 1 to 8 as well as the three sub-domains of domain 9).

The 46 items of domain 10, *basic school skills,* a) *reading,* b) *writing,* c) *mathematics,* d) *physical education,* are not possible for the Ågrenska staff to estimate during the short family course*.* This information is instead collected from the children´s home schools after the course and is therefore not included in the analyses.

For the domains *social and communicative ability, emotions and behaviours, communication and language, gross motor skills, fine motor skills, perception and worldview,* and *prerequisites for learning,* the initial item rates the child’s overall ability within the domain. This is followed by more detailed items rating the child´s abilities in each domain. The overall items are not included in the analyses as the detailed items together are expected to give an estimation of the overall ability. An exception was made for domain 3, *communication and language*. This domain mostly contains items with fixed response options and yes/no items, i.e. no estimations. These items are therefore not included in the analyses. Domain 3 only contains two rating items, the overall one and the more specific *displays interest in communication.* The latter is not included in the analyses due to a great number of missing observations; therefor the overall is included.

Domain 9, *prerequisites for learning*, consists of three sub-domains, *gatherings/group activities, individual work and ability to assimilate information,* all presented separately. Thus totally eleven domains/sub-domains are analysed and presented.

### Interrater reliability

Interrater reliability was calculated for the quantitative items and items with fixed response options, including yes/no answers, a total of 100 items, which are usually observed by Ågrenskas´s educators during the course. This is a larger number of items than what is used for the statistical analyses, chosen in order to get a basis as broad as possible. Three children, with three different diagnoses, were observed during family stays. An additional three children, all with the same diagnosis, were observed during a respite stay at Ågrenska. On each occasion two educators observed the same child. Five different educators, the total number of educators responsible for observations during family courses, carried out the observations. Interrater reliability reached 91%, see Additional file 5.

### Descriptive statistics

Additional file 4 displays frequencies, means, and standard deviations for each of the eleven domains/subdomains subdivided by diagnosis and gender. A child’s score in each domain was constructed by averaging the observer’s ratings of that child across the domain’s items. The potential range of the means in Additional file 4 is thus from 1 (reflecting no difficulties) to 4 (reflecting severe difficulties). One exception is the domain *fine motor skills,* where the observer made four yes (1) or no (0) decisions about problem presence. Here we instead summed up the observer’s choices, which yields a score for each child that ranges from 0 (no problems recognized) to 4 (four problems recognized). The rationale is to simplify interpretation of results. The mean for fine motor skills is thus interpreted straightforward as the mean number of fine motor problems recognized. Another exception is *communication and language* that is a one-item measure.

### Statistical analyses: Factor analysis and internal scale consistency

Eleven domains/subdomains were included in a principal-component analysis, using varimax rotation and Kaiser criterion for deciding on the number of factors. As seen in Table 1, the rotated matrix gave two factors, together explaining 80% variance.

**Table 1:** Principal Component Analysis of the eleven domains/subdomains

Domain/subdomain	Component	
	Social and cognitive self-regulation	Mastery of body and daily activities
Individual work	.873	
Gathering/group activities	.867	
Ability to assimilate information	.858	
Emotions and behaviour	.857	
Ability to manage his/her disability and everyday life	.845	
Social and communicative ability	.779	.498
Communication and language	.642	.565
Gross motor skills		.843
Fine motor skills		.793
Activities of daily life	.479	.739
Perception	.628	.659

Extraction method: principal component analysis. Rotation method: Varimax with Kaiser normalization

The two factors differentiate clearly within the eleven rating scales. The first factor could be characterized as *Social and Cognitive Self-Regulation* and the second as *Mastery of Body and Daily activities*. Four scales show loadings on both factors: *Social and communicative abilities, Activities of Daily Life, Perception* and *Communication and language*. *Social and communicative abilities* and *Activities of Daily Life* both have higher loadings on one of the two factors, factor one and two, respectively. They thus contribute to the definition of each of the two factors. *Perception* and *Communication and language* show cross-loadings of comparable magnitudes. We chose to keep both in the factor model, since the cross-loadings make sense, given the content of the items making up the scales. For *Perception* e.g. the item “Can adjust muscle force” is clearly a motor aspect of behaviour, whereas “Knows the meaning of present, past and future” refers to cognitive abilities. Also for *Communication and language* both cognitive and motor aspects are involved, such as spoken language and body communication.

The Cronbach alphas for the eleven scales in the component analysis were very good (see Table 2). A caveat is that too high an alpha value could indicate redundancy of item content, though we do not think this is the case, considering the actual items of the three scales with the highest alphas.

**Table 2:** Cronbach’s Alpha for the included domains and subdomains

Domain/subdomain	Number of items	Alpha
Prerequisites for learning		
Individual work	4	.96
Gatherings/group activities	7	.96
Ability to assimilate information	10	.95
Emotion and behaviour	30	.87
Ability to manage his/her disability	11	.80
Social/communicative ability	15	.93
Gross motor skills	16	.93
Fine motor skills	11	.75
Activities of daily life	10	.91
Perception	18	.92

The results of the interrater reliability, the descriptive statistics, the factor analysis and scale internal consistency together give positive answers to research questions 1 (How does the functional impairment affect the children’s and adolescents’ school and everyday situation?) and 2 (How do consequences vary within each diagnosis?). The observations show the general magnitudes of consequences of the functional impairments at the same time as the differences between children with one and the same diagnosis vary considerably.

### Statistical analysis: multinomial regression

A multinomial logistic regression was applied to analyse how well the eleven domains/subdomains would differentiate a reference diagnosis (i.e. narcolepsy) from each of the other diagnoses. The eleven domains/subdomains were used as independent variables and the eight diagnoses each contrasted with narcolepsy as dependent variables. The reason for choosing narcolepsy as a reference group was the fact that narcolepsy was considered a diagnosis less affected in terms of functional levels of the rating scales used (see Additional file 6). It was thus a diagnosis close to typical functioning, at the same time as it comprised a large number of individuals, important for the reliability of the analyses. We should note that this analysis was not the focus of our study, but a description of the discriminating power of the scales could nevertheless be of interest. It should also be noted that the low number of participants in quite a few of the diagnoses in combination with the large set of independent variables means that the results should be interpreted with caution. In addition, we chose to not impute any missing values. The analysis applies listwise deletion and is based on a subsample (*n* = 237) of the full sample described in Additional file 4 (*n* = 267).

Tests of assumptions indicated both overdispersion (1.85 for the Pearson parameter estimate) and underdispersion (0.26 for the Deviance parameter estimate); these differentiate from an ideal value of 1, but do not reach an undesired maximum value of 2 [42]. However, as tests of simplified models yielded similar conclusions, and for reasons of providing one analytical framework (instead of many separate analyses), we decided to rely on and report the multinomial regression analysis despite its potential limitations.

The model summarized in Additional file 6 significantly decreased unexplained variance compared to an intercept–only (i.e. “null” or baseline) model. The goodness-of-fit was still poor according to Pearson χ^2^(1776) = 3290.84, *p* < 0.001, although good according to Deviance χ^2^ (1776) = 459.81, *p* = 1.00. This means that Pearson indicates that the predicted values differ significantly from the observed values, i.e. a bad fit of the model, whereas deviance indicates a good fit between predicted and observed values.

When looking at the likelihood ratio tests of the eleven independent variables, we find that four of them do not contribute significantly to the model overall, viz. *Emotions and behaviours*, *Activities of Daily Life*, *Gatherings/group activities*, and *Individual work* (see Additional file 6, Chi-square).

However, when inspecting the regression coefficients in Additional file 6 we find that the domains/subdomains *Emotions and Behaviours* and *Individual Work* differentiate narcolepsy from achondroplasia (*n* = 18, 95% CI for Odds ratio*:* LL = 1.38; HL = 31,661.83) and from neurofibromatosis type 1 (*n* = 10, 95% CI for Odds ratio*:* LL = 0.01; HL = 0.67), respectively. The latter effect is negative suggesting that compared to narcolepsy neurofibromatosis type 1 *decreases* likelihood of individual work problems. Only two scales did not contribute to define any diagnosis: Activity of Daily Life and Gatherings/Group Activities (see Additional file 7 for a descriptive summary of the results).

By comparing the regression coefficients across different scales it may also be seen that they to some extent overlap and to some extent differ, suggesting the scales have both convergent and discriminant validity. As an example, the scale *Ability to assimilate information* (a central part of the factor *Social and Cognitive Self-Regulation*) differentiates narcolepsy (*n* = 68) from Prader Willi (*n* = 14, 95% CI for Odds ratio*:* LL = 1.97; HL = 526.92) and Williams syndromes (*n* = 39, 95% CI for Odds ratio*:* LL = 3.56; HL = 449.89). These two diagnoses are both associated with an increased likelihood of problems in assimilating information. In contrast, the scale Fine motor skills (a central part of the factor *Mastery of Body and Daily Activities*), while also differentiating narcolepsy from the aforementioned diagnoses, in addition differentiates narcolepsy from another four diagnosis, e.g., Ehlers Danlos syndromes (*n* = 30, 95% CI for Odds ratio*:* LL = 3.81; HL = 172.20). Compared to narcolepsy, Ehlers Danlos syndromes increase likelihood of fine-motor problems.

Note that the multinomial analysis was not a main purpose of the study, since the low number of participants in certain diagnoses present a methodological challenge. However, with this reservation, we think that research question 3 (Is it possible to identify diagnosis-specific features that lead to specific pedagogical and every day consequences?), even if not given an unequivocal positive answer, has received support and definitely proved to be meaningful and warrants further research with a quantitative approach.

## Discussion

Most rare health conditions are syndromes, which means several different symptoms, which can vary in number and severity in different individuals with the same diagnosis. It is important to show that there are common diagnosis-specific features and consequences, but also that consequences, and thus needs, can vary within the same diagnosis.

Medical facts need to be translated into specific educational and every day consequences relevant to each individual [35]. Causes, symptoms and consequences are related and approaches and working methods must be based on these in order to adapt treatment, materials, environment and activities to meet the  individual´s needs and create conditions for optimal learning and participation.

Knowledge of diagnosis-specific consequences needs to be systematically collected. For this purpose, the observation instrument has been developed.

In order to adapt to the individual level, knowledge of the individual child's symptoms, their severity and consequences is also needed, as well as knowledge of personal factors [11] in the individual child, not related to the diagnosis.

The presented observation instrument is primarily intended for teachers and special education teachers. In order to carry out the observations, pedagogical knowledge and experience is required. Knowledge of typical development in the educational, emotional and social areas, as well as experience of working with children with disabilities is also necessary. The observations are intended to be carried out in the children´s ordinary school or preschool as well as during leisure activities, which thus have to be planned so that different abilities are possible to observe. The teachers or special education teachers using the observation instrument have to be familiar with the childrens´ schedule and activities as well as with the instrument, the included domains and items in order to observe abilities when performing their ordinary work with the children. Each domain should be observed repeatedly, i.e. each day of the observation period (which could entail for example a stay, a course or a school-week) and preliminary notes should be taken every day. The final ratings should be made after the course. The responsible observers, i.e. teachers and special education teachers, can also obtain information from other colleagues/professionals working with the children, before making their final ratings.

It is important to emphasize that the observation instrument is not intended for the assessment of individual children, but for knowledge formation about a specific diagnosis on a group-level.

The observations of each child included in the present study were carried out during one week, i.e. a relatively short time. The children were observed in an environment that was new to them, together with children and staff they had not met before. In such a short time, the children may not get the chance to show their full repertoire of abilities. Certain items are not observable, such as some school-related and psychosocial abilities or abilities that need to be observed for an extended period of time in order for them to be reliably estimated. Additional information from the home school is therefore of the utmost importance in order to achieve the most nuanced and complete picture possible. The fact that not all estimates can be made by the same observer and during the same period could be seen as a limitation. At the same time, this could also be an advantage, as information is obtained from two different sources and in more than one context.

As most rare diagnoses are syndromes, varying combinations and varying severity of symptoms result in different combinations of consequences within each diagnosis [35]. Therefore, there may be diagnosis-specific consequences, but not a diagnosis-specific total pedagogy, which is suitable in all situations for all individuals with the same diagnosis.

Specifically, the constructed observation instrument should answer the following questions: (1) How does the functional impairment affect the children’s and adolescents’ school and everyday situation? (2) How do consequences vary within each diagnosis? (3) Is it possible to identify diagnosis-specific features that lead to specific every day and pedagogical consequences?

The total number of observations for each of the first three domains and the domains/subdomains five to eleven is between 265 and 267 (see Additional file 4). The fourth scale, *ability to manage his/her disability and everyday life*, contains 243 observations due to rephrasing or addition of totally four items in the latest version of the observation form. Ratings made according to earlier versions thus lack this information.

Narcolepsy was chosen as reference diagnosis as it comprises a large number of participants, necessary for the reliability of the analyses. It was also considered a diagnosis close to typical functioning. The results show that it is the closest to typical functioning in four domains, *communication and language, activities for daily life, ADL gross* and *fine motor skills*, and the second to closest for the other seven domains/subdomains.

As the interrater reliability and Cronbach alphas are good and the content validation showed that domains and items are relevant we may conclude that the means and standard deviations in Additional file 4 represent and describe relevant consequences and their variations in school and everyday life. The factor analysis also showed good statistical qualities of the instrument. The fact that two factors with completely relevant content accounted for 80% of the variation of the information analyzed is reassuring. Thus the constructed instrument could be considered answering the two first questions: (1) How does the functional impairment affect the children’s and adolescents’ school and everyday situation? (2) How do consequences vary within each diagnosis?

Some examples will be given, focusing on analyses of diagnosis groups with 20 observations or more. Those are achondroplasia (n = 20), Prader Willi syndrome (n = 22), Ehlers Danlos syndrome (n = 32), Williams syndrome (n = 43), fragile X syndrome (n = 44) and narcholepsy (n = 72).

Three of the diagnoses appear to be much affected in most domains and subdomains, i.e. Prader Willi, Williams and fragile X syndrome, all including intellectual and neuropsychiatric impairment. These diagnoses show high means, most prominently for *social and communicative ability*, *communication and language*, *gatherings/group activities, individual work* and *ability to assimilate information*. For fragile X syndrome, the means clearly show gender differences with males being more affected than females.

These findings are also in accordance with Ågrenska´s experience and with diagnose specific information from e.g. Frambu [43–45]. Note that these observations not necessarily are inconsistent with the results from the regression analysis, which failed to substantiate differences between narcolepsy and these three diagnoses with respect to *individual work* and *gatherings/group activities*. A possibility to be considered is that other subdomains account for differentiating these diagnoses from narcolepsy with respect to *individual work* and *gatherings/group activities*. However, limitations of the regression analysis also needs to be considered here such as a lack of power to detect smaller effects due to small subsample sizes in particular.

This means that research question 3 (Is it possible to identify diagnosis-specific features that lead to specific pedagogical and every day consequences?) could not be answered positively without reservations. This is an unavoidable obstacle for a quantitative approach given small expected populations, particularly in a small country like Sweden. However, the results show that it is a worthwhile endeavor, but should be applied with caution.

Individuals with Williams syndrome have difficulties understanding spoken language. Despite this, they can display an elaborated productive spoken language, using words and phrases they often do not understand. They can also display boundlessness. Partly because of this they are usually perceived as social and interested in other people, also towards people they do not know, and viewed as positive and friendly [44, 46]. This is in accordance with our observations. Individuals with Williams syndrome are rated as having the second highest difficulties in social and communicative abilities of the included diagnoses.

For Prader Willi syndrome can be noted a somewhat higher standard deviation for *activities for daily life*, 1.01. In this domain the need for help with the food situation is included. According to our experience there are great individual variations in the children´s handling of their overeating problems, depending on learning from early age, degree of compulsory behaviour, as well as parents´ and other people´s knowledge, understanding and endurance.

For both Williams syndrome and Prader Willi syndrome males show higher means on all our analyzed domains/subdomains than females. This is not described in the information brochures that we refer to in this paper and further research is needed.

Ehlers Danlos syndrome, achondroplasia and narcholepsy come out as the diagnoses that are least affected, but anyhow experience various difficulties.

Ehlers Danlos syndrome is divided into 13 subgroups [47, 48], between which we have not distinguished in our observations. The family courses are directed to all individuals with the syndrome regardless of subtype. The different types have common traits of varying severity and strength, but also different prominent symptoms [47, 48]. This can explain the somewhat higher standard deviation for *individual work* (sd 0.82) and *gathering/group activities* (sd 0.69) compared to the other scales for this diagnosis.

Achondroplasia is the diagnosis closest to typical functioning except for *communication and language, activities of daily life, gross and fine motor skills* and in the analyzed diagnoses. This is due to the physical conditions and in line with what can be expected [49]. Means are 1.01–1.65.

For narcolepsy the most affected domains are *ability to manage his/her disability* (m 1.72), *individual work* (m 1.81) and *ability to assimilate information* (m 1.67). These also show the highest standard deviations, 0.49, 0.61 and 0.48 respectively. These results probably have to do with the children´s tiredness, leading to concentration problems and risk of falling asleep during daytime, also at school [50]. The observed children were all diagnosed with narcolepsy after Pandemrix vaccination and the observations were made within about one to two years after their diagnosis was set. At this early stage, their medication may not have been finally customized for all. This can affect both the level of difficulties and the variation.

The results commented above are in line with what can be expected from Ågrenska´s experienes from working with children with these diagnoses, contacts with their teachers and other professionals and also in accordance with previous research and published information referred to in this paper.

Generally, the standard deviations can be considered quite low, but anyhow indicate individual variations in consequences and their severity. We know that for some rare diagnoses there are quite distinctive genotype – phenotype correlations, e.g. Williams syndrome and fragile X syndrome, i.e. the specific genetic mutation leads to specific similarities in appearance, abilities and behaviour in many individuals with the same diagnosis [51]. Our results indicate that variations within diagnoses could be of interest for further research.

## Conclusions

The number of rare diagnoses, as well as the number of individuals living with a rare diagnosis, are increasing thanks to medical progress. The conditions often lead to consequences in everyday life. Therefore, more and more specific needs will appear and the need for knowledge will increase accordingly, not least among educational professionals. The observation instrument presented in this paper can be used to gather information in a structured way about relevant consequences and their variations on a group level in school and everyday life for children and adolescents with rare diagnoses. This knowledge, together with knowledge about the individuals with the diagnoses, can form the basis for adapting methods and environment to meet the educational needs and create conditions for optimal learning and participation.

## Supplementary Information

**Additional file 1.** The Ågrenska observation instrument.

**Additional file 2.** Changes in the observation instrument.

**Additional file 3.** Content validation.

**Additional file 4.** Descriptive statistics.

**Additional file 5.** Interrater reliability.

**Additional file 6.** Multinomial regression analysis.

**Additional file 7.** Summary of contributions of the domains.

## Data Availability

Anonymised data that support the findings of this study are available from the corresponding author upon reasonable request.
