# Supplementary material for: Consequences of rare diagnoses for education and daily life: development of an observation instrument
Source: Orphanet J Rare Dis. 2022 Apr 12;17:165. doi: 10.1186/s13023-022-02303-y (PMC9004121; doi:10.1186/s13023-022-02303-y)
Supplement: Supplementary file 1 — Additional file 1. The Ågrenska observation instrument. [file 13023_2022_2303_MOESM1_ESM.pdf]

ÅGRENSKA

Additional file 1. Ågrenska Observation Instrument

THE ÅGRENSKA OBSERVATION FORM

For preschool and school staff and others, regarding children with disabilities 4-17 years old.
Assessment of the child´s / student's ability in relation to age.

Name: ______________________________________

Code Key: __________________________________

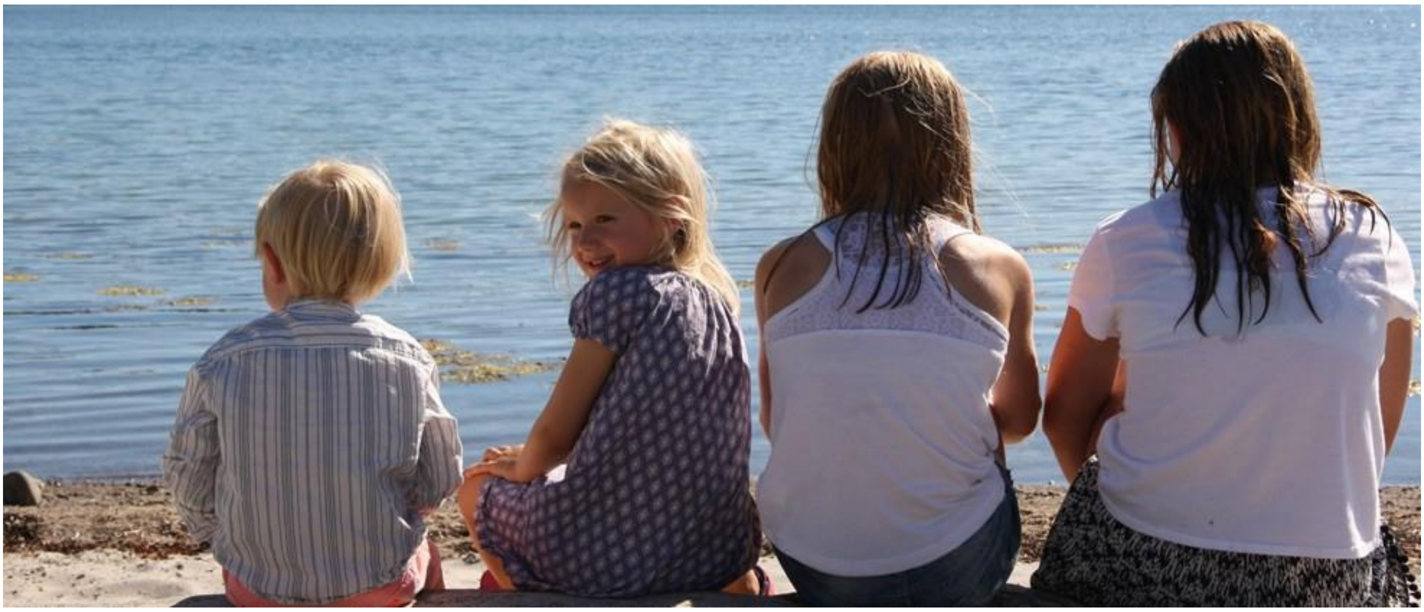

ÅGRENSKA

GENERAL INFORMATION

Id for login is given in the excel document and in the observation form

Diagnosis

- ☐ Albinism
- ☐ Achondroplasia
- ☐ Apert syndrome
- ☐ Asperger syndrome
- ☐ Autoimmune encephalitis
- ☐ Bardet-Biedl syndrome (LMBB)
- ☐ Becker muscular dystrophy
- ☐ Beckwith-Wiedermann syndrome
- ☐ Cat eye syndrome
- ☐ Congenital Disorders of Glycosylation (CDG)
- ☐ Charge syndrome
- ☐ Coffin-Lowry syndrome
- ☐ Costello syndrome
- ☐ Crouzon syndrome
- ☐ Coffin-Siris syndrome
- ☐ Cystinosis
- ☐ Cystic fibrosis
- ☐ Duchenne muscular dystrophy
- ☐ Dystrofia Myotonika type 2
- ☐ Ehlers-Danlos syndrome
- ☐ Ectodermal dysplasia
- ☐ Univentricular heart (UIH)
- ☐ Epidermolysis bullosa
- ☐ Esophageal atresia
- ☐ Phenylketonuria (PKU)
- ☐ Fragile X syndrome
- ☐ Galactosemia
- ☐ Gaucher disease
- ☐ Glutaric aciduria
- ☐ Heriditary spastic paraparesis
- ☐ Hurler disease
- ☐ Ichthyosis
- ☐ Incontinentia pigmenti
- ☐ Jourbert syndrome
- ☐ Kabuki syndrome
- ☐ Klinefelter syndrome
- ☐ Congenital muscular dystrophy
- ☐ Limb-girdle muscular dystrophy
- ☐ Marfan syndrome
- ☐ Marker chromosome 15 syndrome
- ☐ Monosomi 4p syndrome, Wolf-Hirschhorn syndrome
- ☐ Mowat-Wilson syndrome
- ☐ Mucopolysaccaridosis
- ☐ Möbius syndrome
- ☐ Narcolepsy
- ☐ Nemaline myopathy
- ☐ Neurofibromatosis type 1
- ☐ Neurofibromatosis type 2
- ☐ Noonan syndrome
- ☐ Optic nerve hypoplasia
- ☐ Osteogenesis imperfecta
- ☐ Pandas syndrome
- ☐ Dravet syndrome
- ☐ Pompe disease
- ☐ Potocki-Lupski syndrome

☐ Prader Willi syndrome
☐ Silver-Russell syndrome
☐ Sotos syndrome (SOS)
☐ Spina bifida MMC
☐ Sturge-Webers syndrome
☐ Tourette syndrome
☐ Treacher Collin sundrome
☐ Tuberous sclerosis
☐ Turner syndrome
☐ Usher syndrome
☐ VACTERL syndrome
☐ Williams syndrome
☐ Wilson disease
☐ 1p36 deletion syndrome
☐ 22q11 deletion syndrome
☐ Spinal muscular atrophy 1
☐ Spinal muscular atrophy 2
☐ Spinal muscular atrophy 3
☐ Myotonic dystrophy, type 1, congenital onset
☐ Myotonic dystrophy, type 1, childhood onset
☐ Myotonic dystrophy, type 1, adult onset

Gender

☐ Man
☐ Female
☐ Other ______________________________

Age at time of observation

☐ 4 years
☐ 5 years
☐ 6 years
☐ 7 years
☐ 8 years
☐ 9 years
☐ 10 years
☐ 11 years
☐ 12 years
☐ 13 years
☐ 14 years
☐ 15 years
☐ 16 years
☐ 17 years

ÅGRENSKA

The observation made year

- [ ] 2000
- [ ] 2001
- [ ] 2002
- [ ] 2003
- [ ] 2004
- [ ] 2005
- [ ] 2006
- [ ] 2007
- [ ] 2008
- [ ] 2009
- [ ] 2010
- [ ] 2011
- [ ] 2012
- [ ] 2013
- [ ] 2014
- [ ] 2015
- [ ] 2016
- [ ] 2017
- [ ] 2018
- [ ] 2019
- [ ] 2020
- [ ] 2021
- [ ] 2022
- [ ] 2023
- [ ] 2024
- [ ] 2025
- [ ] 2026
- [ ] 2027
- [ ] 2028
- [ ] 2029
- [ ] 2030
- [ ] 2031
- [ ] 2032
- [ ] 2033
- [ ] 2034
- [ ] 2035

Educator in charge

Entered by

Language

- [ ] Swedish
- [ ] English

DAYTIME RESIDENCE

- ☐ Preschool
- ☐ Special preschool
- ☐ Compulsory school including preschool class
- ☐ Compulsory school with special syllabus for children with learning disabilities
- ☐ Special school for children with learning disabilities
- ☐ Training school
- ☐ Upper secondary school
- ☐ Upper secondary school with special syllabus for adolescents with learning disabilities
- ☐ Upper secondary school with special syllabus for adolescents with learning disabilities
- ☐ Training school, upper secondary level
- ☐ Individual program / introduction program
- ☐ Home schooling
- ☐ Hospital school
- ☐ Other ______________________________

Class/Year

____
____
____
____
____

Number of pupils in class/group

____
____
____
____
____

	Yes, completely	No	Yes in part	Not relevant	Data not available
Special education/training	☐	☐	☐	☐	☐
Assistant/resource in preschool/school	☐	☐	☐	☐	☐
Attends full class	☐	☐	☐	☐	☐
Participates in physical education	☐	☐	☐	☐	☐
Individual physical education	☐	☐	☐	☐	☐

Number of staff in class/group

____
____
____
____
____

Special education number of hours per week

____
____
____
____
____

Assistant/Resource number of hours per week

____
____
____
____
____

Attends full class number of hours per week

____
____
____

ÅGRENSKA

DISABILITIES/ SYMPTOMS

	Yes	No	Data not available
Visual impairment	☐	☐	☐
Hearing impairment	☐	☐	☐
Behavioural deviation	☐	☐	☐
Autism spectrum disorder	☐	☐	☐
Epilepsy	☐	☐	☐
Intellectual disability	☐	☐	☐
Motor impairment	☐	☐	☐
Heart disease	☐	☐	☐
Respiratory problems	☐	☐	☐
Allergy /hypersensitivity/ intolerance	☐	☐	☐
Language impairment	☐	☐	☐

Disability / symptom other than above

☐ Data not available

☐ No

☐ Yes ______________________________

Further established diagnosis in addition to main diagnosis

☐ Data not available

☐ No

☐ Yes ______________________________

Aids

☐ Data not available

☐ No

☐ Yes ______________________________

Medication

☐ Data not available

☐ No

☐ Yes ______________________________

Training program

☐ Data not available

☐ No

☐ Yes ______________________________

Special diet

☐ Data not available

☐ No

☐ Yes ______________________________

Other, what

☐ No

☐ Yes ______________________________

INFORMATION FROM SCHOOL AT PLACE OF RESIDENCE.
Before the course

Give examples of things that work well in preschool/school

What are the child's interests and what does he/she spontaneously choose to work on?

In your experience, what difficulties does the child encounter in preschool/ school situations?

What do you do to reduce these difficulties?

How does the child/pupil work socially in the group?

ÅGRENSKA

1. SOCIAL AND COMMUNICATIVE ABILITY

How do you assess the child's/student's social and communicative ability in relation to age?

	No problems	Mild problems	Moderate problems	Servere problems	Data not available
Agrenska	☐	☐	☐	☐	☐
School	☐	☐	☐	☐	☐

	No problems	Mild problems	Moderate problems	Servere problems	Cannot assess	Data not available
Makes contact with peers	☐	☐	☐	☐	☐	☐
Responds to contact with peers	☐	☐	☐	☐	☐	☐
Makes contact with adults	☐	☐	☐	☐	☐	☐
Responds to contact with adults	☐	☐	☐	☐	☐	☐
Has adequate eye contact	☐	☐	☐	☐	☐	☐
Shows that he/she recognizes other people	☐	☐	☐	☐	☐	☐
Plays/socializes with others	☐	☐	☐	☐	☐	☐
Plays/socializes with others without help from adults	☐	☐	☐	☐	☐	☐
Can participate in free play at break time	☐	☐	☐	☐	☐	☐

Agrenska: Give your general impression of the social and communicative ability

Agrenska: In what situations does it work best?

School: Give your general impression of the social and communicative ability

School: In what situations does it work best?

ÅGRENSKA

2. EMOTIONS AND BEHAVIOURS

How do you assess the child's/student's emotional ability or behaviour in relation to age?

	No problems	Mild problems	Moderate problems	Servere problems	Data not available
Agrenska	☐	☐	☐	☐	☐
School	☐	☐	☐	☐	☐

	Not at all	Some	A fair amount	To a high degree	Cannot assess	Data not available
Behaves boundless	☐	☐	☐	☐	☐	☐
Behaves dissatisfied	☐	☐	☐	☐	☐	☐
Displays signs of anxiety/worry	☐	☐	☐	☐	☐	☐
Behaves restlessly	☐	☐	☐	☐	☐	☐
Is impulse controlled	☐	☐	☐	☐	☐	☐
Has fast, unmotivated, mood swings	☐	☐	☐	☐	☐	☐
Displays a lack of confidence	☐	☐	☐	☐	☐	☐
Displays a lack of empathic ability	☐	☐	☐	☐	☐	☐

Agrenska: Give your general impression of the emotional behaviour

____
____
____
____
____

School: Give your general impression of the emotional behaviour

____
____
____
____
____

Agrenska: Other observable behaviors

____
____
____
____
____

School:Other observable behaviours

____
____
____
____
____

ÅGRENSKA

3. COMMUNICATION AND LANGUAGE

How do you assess the child's/student's linguistic and communicative ability in relation to age?

	No problems	Mild problems	Moderate problems	Severe problems	Data not available
Agrenska	☐	☐	☐	☐	☐
School	☐	☐	☐	☐	☐

Speech

- ☐ Lacking speech
- ☐ Speech which is difficult to understand
- ☐ Somewhat unclear speech
- ☐ Stutters
- ☐ Difficulty expressing him/herself/explaining/finding the right word
- ☐ Difficulty understanding the speech of others
- ☐ Cannot assess
- ☐ Data not available

Use of speech

- ☐ Response sounds
- ☐ Single words
- ☐ Two-word sentences
- ☐ Multi-word sentences
- ☐ Cocktail speech
- ☐ Echo speech
- ☐ Cannot assess
- ☐ Data not available

Use of support or alternative to speech

- ☐ Body language
- ☐ Signs as support
- ☐ Sign language
- ☐ Concrete objects
- ☐ Photographs
- ☐ Drawings
- ☐ Bliss
- ☐ Written words
- ☐ Cannot assess
- ☐ Data not available

Use of technical communication aids

- ☐ Data not available
- ☐ No
- ☐ Yes, which ______________________________

Displays interest in communication

- ☐ To a high degree
- ☐ To a relatively high degree
- ☐ Some
- ☐ Not at all
- ☐ Cannot assess
- ☐ Data not available

Displays communicative and linguistic ability only in specific situations

- ☐ Data not available
- ☐ No
- ☐ Yes, how ______________________________

Displays communicative and linguistic ability only together with certain people

- ☐ Data not available
- ☐ No
- ☐ Yes, how ______________________________

Agrenska: Additional info, communication / language

School: Additional info, communication / language

ÅGRENSKA

4. ABILITY TO HANDLE HIS/HER DISABILITY AND HIS/HER EVERYDAY LIFE

In relation to the child's/student's age

	To a high degree	To a relatively high degree	To some degree	Not at all	Cannot assess	Data not available
Displays a connection with peers	☐	☐	☐	☐	☐	☐
Displays a connection with family	☐	☐	☐	☐	☐	☐
Displays a connection with others	☐	☐	☐	☐	☐	☐
Tries to solve occurring everyday problems	☐	☐	☐	☐	☐	☐
Seems to have a general well-being, seems satisfied	☐	☐	☐	☐	☐	☐
Shows a positive attitude to his/her surroundings	☐	☐	☐	☐	☐	☐
Shows trust in people in his/her surroundings	☐	☐	☐	☐	☐	☐
Is familiar with/knows about his/her disability	☐	☐	☐	☐	☐	☐
Is able to describe his/her disability	☐	☐	☐	☐	☐	☐

Agrenska: Additional info, ability to cope with his/her disability and his/her everyday life

____

____

____

____

____

School: Additional info, ability to cope with his/her disability and his/her everyday life

____

____

____

____

____

5. ADL

In relation to the child's/student's age

Needs help with clothes

- [ ] Not at all
- [ ] Somewhat
- [ ] To a relatively high degree
- [ ] To a high degree
- [ ] Data not available

Needs and aids regarding clothing

Needs help with the food situation

- [ ] Not at all
- [ ] Somewhat
- [ ] To a relatively high degree
- [ ] To a high degree
- [ ] Data not available

Needs and aids regarding the food situation

Needs help with general hygiene

- [ ] Not at all
- [ ] Somewhat
- [ ] To a relatively high degree
- [ ] To a high degree
- [ ] Data not available

Needs and aids regarding general hygiene

Needs help when going to the toilet

- [ ] Not at all
- [ ] Somewhat
- [ ] To a relatively high degree
- [ ] To a high degree
- [ ] Data not available

Needs and aids when going to the toilet

Agrenska: Additional info ADL

School: Additional info ADL

ÅGRENSKA

6. GROSS MOTOR SKILLS

How do you assess the child's/student's gross motor skills in relation to age?

	No problems	Mild problems	Moderate problems	Severe problems	Data not available
Agrenska	☐	☐	☐	☐	☐

	No problems	Mild problems	Moderate problems	Severe problems	Cannot assess	Data not available
Balance sitting	☐	☐	☐	☐	☐	☐
Balance standing	☐	☐	☐	☐	☐	☐
Agility	☐	☐	☐	☐	☐	☐
Ability to walk	☐	☐	☐	☐	☐	☐
Ability to stand	☐	☐	☐	☐	☐	☐
Ability to run	☐	☐	☐	☐	☐	☐
Ability to jump	☐	☐	☐	☐	☐	☐
Ability to walk in nature	☐	☐	☐	☐	☐	☐
Ability to climb stairs	☐	☐	☐	☐	☐	☐
Ability to plan, execute and coordinate movements	☐	☐	☐	☐	☐	☐

	Not at all	Somewhat	To a relatively high degree	To a high degree	Cannot assess	Data not available
Deviating movements/ movement patterns	☐	☐	☐	☐	☐	☐
Deviating muscle tone	☐	☐	☐	☐	☐	☐

Gross motor aids

____

Agrenska: Additional info, gross motor skills

____

School: Additional info, gross motor skills

____

7. FINE MOTOR SKILLS

How do you assess the child's/student's fine motor skills in relation to age?

	No problems	Mild problems	Moderate problems	Severe problems	Data not available
Agrenska	☐	☐	☐	☐	☐

	Yes	No	Cannot assess	Not relevant	Data not available
Is able to cut using scissors	☐	☐	☐	☐	☐
Is able to snap buttons	☐	☐	☐	☐	☐

	No	Yes	Cannot assess	Not relevant	Data not available
Has immature/unusual pen grip	☐	☐	☐	☐	☐
Has difficulty handling small objects	☐	☐	☐	☐	☐

Hand preference

☐ Is right handed/prefers right hand

☐ Is left handed/prefers left hand

☐ Lacks hand preference

☐ Cannot assess

☐ Data not available

Agrenska: Additional info, fine motor skills (e.g. if the child uses support, orthoses, prosthetics, specially made tools, specially made grip for pen/scissors etc, to perform hands-on activities)

____

____

____

____

____

School: Additional info, fine motor skills (e.g. if the child uses support, orthoses, prosthetics, specially made tools, specially made grip for pen/scissors etc, to perform hands-on activities)

____

____

____

____

____

8. PERCEPTION AND WORLDVIEW

How do you assess the child's/student's perceptual ability in relation to age?

	No problems	Mild problems	Moderate problems	Severe problems	Data not available
Agrenska	☐	☐	☐	☐	☐

	To a high degree	To a relatively high degree	To some degree	Not at all	Cannot assess	Data not available
Has body image	☐	☐	☐	☐	☐	☐
Has expected feeling for touch	☐	☐	☐	☐	☐	☐
Has expected feeling for pain	☐	☐	☐	☐	☐	☐
Has eye-hand coordination	☐	☐	☐	☐	☐	☐
Has eye-foot coordination	☐	☐	☐	☐	☐	☐
Can adapt muscle power	☐	☐	☐	☐	☐	☐
Can copy the movements of others	☐	☐	☐	☐	☐	☐
Can judge distance	☐	☐	☐	☐	☐	☐
Can handle height differences	☐	☐	☐	☐	☐	☐
Can recognise everyday sounds	☐	☐	☐	☐	☐	☐
Can locate sound source	☐	☐	☐	☐	☐	☐
Can locate him/herself in his/her immediate vicinity	☐	☐	☐	☐	☐	☐

	To a high degree	To a relatively high degree	To some degree	Not at all	Cannot assess	Not applicable	Data not available
Knows the meaning of morning/evening	☐	☐	☐	☐	☐	☐	☐
Knows the meaning of present/past/future	☐	☐	☐	☐	☐	☐	☐
Can tell time mechanically	☐	☐	☐	☐	☐	☐	☐
Understands the meaning of a given time	☐	☐	☐	☐	☐	☐	☐
Is able to plan his/her time	☐	☐	☐	☐	☐	☐	☐

Structures his/her day/time with the help of

- ☐ Concrete objects
- ☐ Photographs
- ☐ Drawings
- ☐ Bliss
- ☐ Written words
- ☐ Cannot assess
- ☐ No data available

Agrenska: Additional info, perception and worldview

____
____
____
____
____

School: Additional info, perception and worldview

____
____
____
____
____

ÅGRENSKA

9. PREREQUISITES FOR LEARNING

How do you assess the child's/ student's ability to learn in relation to age?

	No problems	Mild problems	Moderate problems	Severe problems	Data not available
Agrenska	☐	☐	☐	☐	☐
School	☐	☐	☐	☐	☐

a) Gatherings/group activities

	No problems	Mild problems	Moderate problems	Severe problems	Cannot asses	Data not available
Shows interest	☐	☐	☐	☐	☐	☐
Participates actively	☐	☐	☐	☐	☐	☐
Participates without disturbing	☐	☐	☐	☐	☐	☐
Manages without an adult next to him/her	☐	☐	☐	☐	☐	☐
Can handle group size >15 people	☐	☐	☐	☐	☐	☐
Can handle group size 5-15 people	☐	☐	☐	☐	☐	☐
Can handle group size <5 people	☐	☐	☐	☐	☐	☐

Agrenska: Additional info, gathering

____

School: Additional info, gathering

____

b) Individual work

	To a high degree	To a relatively high degree	To some degree	Not at all	Cannot assess	Data not available
Displays ability to concentrate	☐	☐	☐	☐	☐	☐
Works independently	☐	☐	☐	☐	☐	☐
Is able to start, execute and finish his/her tasks	☐	☐	☐	☐	☐	☐
Is able to organize his/her work	☐	☐	☐	☐	☐	☐

Agrenska: Other individual work

____

School: Other individual work

____

c) Ability to assimilate information

	To a high degree	To a relatively high degree	To some degree	Not at all	Cannot assess	Data not available
Understands and can use oral information	☐	☐	☐	☐	☐	☐
Remembers given information	☐	☐	☐	☐	☐	☐
Shows motivation	☐	☐	☐	☐	☐	☐
Shows initiative	☐	☐	☐	☐	☐	☐
Shows attentiveness	☐	☐	☐	☐	☐	☐

	Not at all	To some degree	To a relatively high degree	To a high degree	Cannot assess	Data not available
Has trouble getting started	☐	☐	☐	☐	☐	☐
Needs repetition	☐	☐	☐	☐	☐	☐
Needs concrete materials	☐	☐	☐	☐	☐	☐
Needs one-to-one tuition	☐	☐	☐	☐	☐	☐

	To a high degree	To a relatively high degree	To some degree	Not at all	Cannot assess	Not applicable	Data not available
Understands and can use written information	☐	☐	☐	☐	☐	☐	☐

Agrenska: Additional info, ability to make use of information

____
____
____
____
____

School: Additional info, ability to make use of information

____
____
____
____
____

10. BASIC SCHOOL SKILLS.

Information from home school

a) Reading ability

	No problems	Mild problems	Moderate problems	Severe problems	Data not available
How do you assess the child's/student's reading ability in relation to age?	☐	☐	☐	☐	☐

Can sound

- ☐ Yes
- ☐ No
- ☐ Partly
- ☐ Cannot assess/not applicable
- ☐ Comment can sound ______________________________

Knows all sounds/phonemes

- ☐ Yes
- ☐ No
- ☐ Partly
- ☐ Cannot assess/not applicable
- ☐ Comment all sounds/phonemes

Can read

- ☐ Yes
- ☐ No
- ☐ Partly
- ☐ Cannot assess/not applicable
- ☐ Comment can read ______________________________

Has reading comprehension

- ☐ Yes
- ☐ No
- ☐ Partly
- ☐ Cannot assess/not applicable
- ☐ Comment has reading comprehension ______________________________

Likes to read

- ☐ Yes
- ☐ No
- ☐ Partly
- ☐ Cannot assess/not applicable
- ☐ Comment likes to read ______________________________

Reads slowly

- ☐ Yes
- ☐ No
- ☐ Partly
- ☐ Cannot assess/not applicable
- ☐ Comment reads slowly ______________________________

Switches sounds

- ☐ Yes
- ☐ No
- ☐ Partly
- ☐ Cannot assess/not applicable
- ☐ Comment switches sounds ______________________________

Guesses

- ☐ Yes
- ☐ No
- ☐ Partly
- ☐ Cannot assess/not applicable
- ☐ Comment guesses ______________________________

Additional info, reading skills

____
____
____
____
____

b) Writing skills

	No problems	Mild problems	Moderate problems	Severe problems	Data not available
How do you assess the children's/student´s writing skills in relation to age	☐	☐	☐	☐	☐

Can spell

- ☐ Yes
- ☐ No
- ☐ Partly
- ☐ Cannot assess/not applicable
- ☐ Comment can spell ______________________________

ÅGRENSKA

Can form letters/write neatly

- ☐ Yes
- ☐ No
- ☐ Partly
- ☐ Cannot assess/not applicable
- ☐ Comment can spell ______________________________

Can express him/herself in writing in an age-appropriate way

- ☐ Yes
- ☐ No
- ☐ Partly
- ☐ Cannot assess/not applicable
- ☐ Comment can express him/herself in writing in an age-appropriate way ______________________________

Additional info, writing skills

____
____
____
____
____

c) Mathematical skills

	No problems	Mild problems	Moderate problems	Severe problems	Data not available
How do you assess the child´s/student´s mathematical skill in relation to age?	☐	☐	☐	☐	☐

Knows addition

- ☐ Yes
- ☐ No
- ☐ Partly
- ☐ Cannot assess/not applicable
- ☐ Comment knows addition ______________________________

Knows subtraction

- ☐ Yes
- ☐ No
- ☐ Partly
- ☐ Cannot assess/not applicable
- ☐ Comment knows subtraction ______________________________

Knows multiplication

- ☐ Yes
- ☐ No
- ☐ Partly
- ☐ Cannot assess/not applicable
- ☐ Comment knows multiplication ______________________________

Knows division

- ☐ Yes
- ☐ No
- ☐ Partly
- ☐ Cannot assess/not applicable
- ☐ Comment knows division ______________________________

Can handle problem solving

- ☐ Yes
- ☐ No
- ☐ Partly
- ☐ Cannot assess/not applicable
- ☐ Comment can handle problem solving ______________________________

ÅGRENSKA

Has mathematical awareness

- ☐ Yes
- ☐ No
- ☐ Partly
- ☐ Cannot assess/not applicable
- ☐ Comment has mathematical awareness ______________________________

Understands and is able to use units

- ☐ Yes
- ☐ No
- ☐ Partly
- ☐ Cannot assess/not applicable
- ☐ Comment understands and is able to use units ______________________________

Additional info, mathematical skills

d) Physical education.
How do you assess the student's ability in physical education in relation to age?

- ☐ No problems
- ☐ Mild problems
- ☐ Moderate problems
- ☐ Severe problems
- ☐ Cannot assess/not applicable
- ☐ Data not available

Has trouble/does not like to participate in teamsports, eg. football, rounders, floorball

- ☐ Yes
- ☐ No
- ☐ Partly
- ☐ Cannot assess/not applicable
- ☐ Comment/example ______________________________

What steps do you take to make physical education accessible to the student?

____
____
____
____
____
