# Supplementary material for: Consequences of rare diagnoses for education and daily life: development of an observation instrument
Source: Orphanet J Rare Dis. 2022 Apr 12;17:165. doi: 10.1186/s13023-022-02303-y (PMC9004121; doi:10.1186/s13023-022-02303-y)
Supplement: Supplementary file 2 — Additional file 2. Changes in the observation instrument. [file 13023_2022_2303_MOESM2_ESM.pdf]

Additional file 2. Changes in the Ågrenska observation instrument from original version to final version

Domain	Original version 2000	Changes made 2008	Changes made 2014	Final version 2018
Cover page, background information	Individual integration in compulsory school	Compulsory school with special syllabus for children with learning disabilities	NA	Added:<br>preschool, home schooling, hospital school
	Individual integration in upper secondary school	Upper secondary school with special syllabus for adolescents with learning disabilities	NA	
	Class	Year	NA	Added:<br>Attends full class, number of hours per week
	General information<br>yes, no, slight, moderate, severe	Disabilities/Symptoms<br>Yes, no		
	Mental retardation	Mental retardation<br>Autism added		Intellectual disability<br>Allergy/hypersensitivity added
Social and Communicative ability	Same 2000 and 2008	Same 2000 and 2008	Domain name changed to Social *and Communicative* ability<br>Added item:<br>In what situations does it work best?	

Emotions and Behaviours	Same 2000 and 2008	Same 2000 and 2008		*Has* low confidence changed to displays a lack of confidence<br>*Has* a lack of empathic ability changed to *Displays* a lack of empathic ability
Observable Behaviours	Same 2000 and 2008	Same 2000 and 2008	The item *Involuntary movement* moved to the Domain *Gross Motor skills*	
Activities of Daily Life (ADL)	Same 2000 and 2008	Same 2000 and 2008		
Communication and Language	Same 2000 and 2008	Same 2000 and 2008	Added items:<br>Shows interest in communication<br>Shows communicative and linguistic ability only in certain situations<br>Shows communicative and linguistic ability only with certain persons	Added items:<br>Shows difficulties expressing him/herself, finding the right word
Ability to handle his/her disability and his/her everyday life	Same 2000 and 2008	Same 2000 and 2008	Removed items:<br>Has a positive outlook on life and has a sense of coherence<br>Has control over his/her life, seems to accept his/her disability	Removed items:<br>Avoids everyday problems

			Has trust changed to shows trust in people around him/her<br><br>Added item:<br>Shows a positive attitude to his/her environment	
Gross Motor skills	Same 2000 and 2008	Same 2000 and 2008	Removed:<br>Head control	High, low, changing muscle tonus changed to atypical muscle tonus<br>Involuntary movement changed to atypical movement/movement pattern
Fine Motor skills	Same 2000 and 2008	Same 2000 and 2008	Removed:<br>Can reach for an object	
Perception and World view	Same 2000 and 2008	Same 2000 and 2008		Is hypersensitive to touch changed to Has expected feeling for touch<br><br>Has an unusually high pain threshold changed to Has expected feeling for pain
Prerequisites for Learning<br>• Individual work	Same 2000 and 2008	Same 2000 and 2008		Has ability to concentrate changed to shows ability to concentrate

• Ability to assimilate information				Has motivation changed to shows motivation, attention, initiative<br><br>Added:<br>Understands and can assimilate written information
• Reading ability	Same 2000 and 2008	Same 2000 and 2008		Added:<br>Knows all sounds,<br>Reverses sounds<br><br>Removed:<br>Reverses words
• Writing skills				Understands and can assimilate written information moved to ability to assimilate information<br><br>Removed:<br>Reverses letters
