# Supplementary material for: Consequences of rare diagnoses for education and daily life: development of an observation instrument
Source: Orphanet J Rare Dis. 2022 Apr 12;17:165. doi: 10.1186/s13023-022-02303-y (PMC9004121; doi:10.1186/s13023-022-02303-y)
Supplement: Supplementary file 3 — Additional file 3. Content validation. [file 13023_2022_2303_MOESM3_ESM.pdf]

Additional file 3. Content validation of items in the Ågrenska observation instrument against other instruments or assessment tools

Domain name in the Ågrenska observation instrument	Items in the Ågrenska observation instrument	Equivalent item in another instrument/assessment tool	Name of other instrument/assessment tool
SOCIAL AND COMMUNICATIVE ABILITY			
	Makes contact with peers<br>Responds to contact with peers	Is the child/pupil interested in socializing with other children/pupils or does he/she rather play alone?<br>Does the child prefer to socialize with children of the same age or younger/older children?	The Stockholm material (Stockholmsmaterialet), Social interaction with peers and adults
	Makes contact with adults<br>Responds to contact with adults	Does the child/pupil rather turn to adults or peers?	The Stockholm material (Stockholmsmaterialet), Social interaction with peers and adults
	Has adequate eye contact	Has poor eye contact<br>Has a clearly deviating ability to make eye contact	Vineland, Domain Maladaptive Behavior<br>5-15 Questionnaire, Domain Social Competence
	Shows that he/she recognizes other people	Reaches for familiar person	Vineland, Domain Socialization
	Plays/socializes with others<br>Plays/socializes with others without help from adults	Plays simple interaction games with others<br>Participates in at least one	Vineland, Domain Socialization

		game with others<br>Follows rules in simple games without being reminded	
	Can participate in free play at break time	How does the pupil function in free activities?	Vineland, Domain Maladaptive Behaviour<br>5-15 Questionnaire, Domain Social Competence<br>The Gotland material (Gotlandsmaterialet)
EMOTIONS AND BEHAVIOURS			
	Behaves boundless	Often interrupts or intrudes<br>Was boundless	5-15 Questionnaire, Domain Impulsivity<br>ASSQ-form
	Behaves dissatisfied	Is stubborn or sullen<br>Is often angry and resentful<br>Is often touchy or easily annoyed by others<br>Often argues with adults	Vineland, Domain Maladaptive Behaviour<br>SNAP IV
	Displays signs of anxiety/worry	Seems tense and complains of nervousness<br>Exhibits extreme anxiety	5-15 Questionnaire, Domain Psychological Symptoms<br>Vineland, Domain Maladaptive Behaviour
	Behaves restlessly	Is constantly in motion in some way, has difficulty sitting still on the chair	5-15 Questionnaire, Domain Executive Function
	Is impulse controlled	Is too impulsive	Vineland, Domain Maladaptive Behaviour

		Often blurts out answers before questions have been completed<br>Often interrupts or intrudes on others<br>Often has difficulty awaiting his/her turn	SNAP IV
	Has fast, unmotivated, mood swings	Fast and sharp mood swings	Conners scale
		Often loses his/her temper	SNAP IV
		Cries or laughs too easily	Vineland, Domain Maladaptive Behaviour
	Displays a lack of confidence	Has low self-esteem	5-15 Questionnaire, Domain Social Competence
	Displays a lack of empathic ability	Has difficulty perceiving/understanding how other people feel	5-15 Questionnaire, Domain Social Competence
COMMUNICATION AND LANGUAGE			
	Somewhat unclear speech<br>Stutters<br>Difficulty expressing him/herself/explaining/finding the right word<br>Difficulty understanding the speech of others	Speaks unclearly<br>Stutters<br>Has difficulty finding the right words or paraphrases<br>Has difficulty speaking fluently<br>Have difficulty understanding explanations and instructions<br>Having trouble understanding what someone said<br>Often mishears, misinterprets words	5-15 Questionnaire, Domain Language

Lacking speech<br>Speech which is difficult to understand	Lacking Speech<br>Speech which is difficult to understand	MunH observation form
Use of speech:		
Response sounds	Makes cooing, gurgling or other sounds when spoken to or fondled by caregiver	Vineland, Domain Communication
Single words<br>Two-word sentences, multi-word sentences	Uses X number (2-20) clear words<br>Combines two or more words into short phrases	Griffiths, Language subscale
Cocktail speech	Spoke without a sense of meaning and content (learned, "cocktail talk", repeated questions)	ASSQ A-form
Echo speech	"Echo speech, ie uses words but lacks meaning and understanding"	Autism and communication, degree project
Use of support or alternative to speech:	Description of AAC:	
Body language	"… signals…., Gestures or body movements that convey a certain meaning"	Autism and communication, degree project
Sign as support		

Sign language<br>Concrete objects<br>Photographs<br>Drawings<br>Bliss<br>Written words	"…signs as support/signing with speech…"<br>"Sign language…"<br>"…object..."<br>"…photographs…"<br>"…pictures…"<br>"…bliss…"<br>"…words/letters…"	
	How does the student communicate verbally/non-verbally, e.g. signs, body language, pictures?	The Stockholm material (Stockholmsmaterialet), Communication
Displays an interest in communication	Appreciates picture book, looks, points, flips<br>"Talking" to people around him/her<br>Pays attention to shouts	Griffiths, Language subscale
Displays communicative and linguistic ability only in specific situations	Have not found the equivalent, but observed in several conditions	
Displays communicative and linguistic ability only together with certain people	Have not found the equivalent, but observed in several conditions	

ABILITY TO HANDLE HIS/HER DISABILITY AND HIS/HER EVERYDAY LIFE			
	Displays a connection with peers	Think about the people you meet on a daily basis, apart from those closest to you. How well do you know them?<br>Think of them as strangers…know them very well	SOC
	Displays a connection with family	Not found anywhere else. Interesting as family members are usually the people you develop relationships with early in life	
	Displays a connection with others	Think about the people you meet on a daily basis, apart from those closest to you. How well do you know them?<br>Think of them as strangers…know them very well	SOC
	Tries to solve occurring everyday problems	When faced with a difficult problem, the solution is:<br>Always confusing, hard to find ...completely clear<br>Which statement best describes how you view life? It is always possible to find a solution to	SOC

	life's diffulties...there is no solution	
Seems to have a general well-being, seems satisfied<br>Shows a positive attitude to his/her surroundings	Life is:<br>completely interesting …completely boring<br>When you think about your life, do you often: Feel how wonderful it is to be alive...ask yourself why you exist<br>Do you feel unfairly treated? very often...very rarely/never	SOC
Shows trust in people in his/her surroundings	Have people you trusted disappointed you? Has never happened … has happened often<br>Do you think that in the future there will always be people you can count on? You are absolutely sure of that …you doubt it	SOC
Knows /knows about his/her disability<br>Can describe his/her disability	Not found anywhere else. Knowing and describing your disability is important to be able to manage your everyday life, understand yourselves and your needs in relation to others and to be able to influence your situation. This position is based	

		on experiences from Ågrenska’s focus group interviews with adults who have rare conditions.	
ACTIVITIES OF DAILY LIFE (ADL)			
	Clothes	From can put on a hat or mittens to dress completely and tie shoes	Griffiths, Personal-Social subscale
	The food situation	From helping to set the table, be active and manage most things during a meal to cutting and eating with a knife and fork	Griffiths, Personal-Social subscale
	General hygiene	From hand washing to washing your hair by yourself	Griffiths, Personal-Social subscale
	Visits to the toilet	Bowel and bladder control	Griffiths, Personal-Social subscale
GROSS MOTOR SKILLS			
	Balance sitting	Sits supported, sits unsupported for at least one minute	Vineland, Domain Motor Skills
	Balance standing	Can stand on one leg, can walk on chalk lines	Griffiths, Locomotor subscale

Agility	Can run at a steady speed and running style<br>Difficult to run fast and smooth<br>Clumsy and inflexible in their movements	Griffiths, Locomotor subscale<br><br>5-15 Questionnaire, Domain Gross motor skills
Ability to stand<br>Ability to walk<br>Ability to run<br><br>Ability to jump	Can stand without support<br>Can walk well on even ground<br>Can run at a steady speed and running style<br>Jumping with both feet together<br>Jumps from steps	Griffiths, Locomotor subscale
Ability to walk in nature	Walks outdoors on uneven ground, on bumpy ground	PEDI, Domain Motor ability
Ability to climb stairs	Walks up / down stairs with one foot on each step without support	Griffiths, Locomotor subscale
Ability to plan, execute and coordinate movements	Can play hopscotch and push a block in front with the same foot	Griffiths, Locomotor subscale
Deviating moments/movement patterns<br>Deviating muscle tone	What is the pupil's posture and muscle tone, symmetrical movements and differences between the body halves when the pupil performs a movement?	The Stockholm material (Stockholmsmaterialet), Motor ability

FINE MOTOR SKILLS			
	Is able to cut using scissors	Cuts using scissors	Griffiths, Eye-and-Hand Coordination subscale
	Is able to snap buttons	Can snap buttons<br>Has difficulties snapping buttons	Griffiths, Personal-Social subscale<br>5-15 Questionnaire, Domain Finmotorik
	Has immature/unusual pen grip	Has immature pen grip, holds the pen in an unusual way	5-15 Questionnaire, Domain Fine<br>motor skills
	Has difficulty handling small objects	Has difficulty picking, assembling and handling small items	5-15 Questionnaire, Domain Fine motor skills
	Is right handed/prefers right hand<br>Is left handed/prefers left hand	Not found anywhere else	
	Lacks hand preference	Has not developed hand dominance	5-15 Questionnaire, Domain Fine motor skills
PERCEPTION AND WORLD VIEW			
	Has body image	Has insecure body image	5-15 Questionnaire, Domain Perception
	Has expected feeling for touch	Is hypersensitive to touch	5-15 Questionnaire, Domain Perception

Has expected feeling for pain	Reacts surprisingly little to cold, pain, etc.	5-15 Questionnaire, Domain Perception
Has eye-hand coordination	How does the child/pupil coordinate movements such as eye-hand, eye-foot, right and left hand?	The Stockholm material (Stockholmsmaterialet), Motor skills
Has eye-foot coordination	Can play hopscotch and push a block in front with the same foot	Griffiths, Locomotor subscale
Can adapt muscle power	Has difficult to use a pencil, trembles, presses too hard	5-15 Questionnaire, Domain Fine motor skills
Can copy the movements of others	Has difficulty imitating other people's movements, for example in singing games	5-15 Questionnaire, Domain Fine motor skills
Can judge distance	Has difficulty assessing distance and size ratios	5-15 Questionnaire, Domain Perception
Can handle height differences	Becomes insecure at height differences such as climbing stairs, takes support from handrails, walls, etc.	5-15 Questionnaire, Domain Perception
Can locate sound source	Searching for sound source with head movements	Griffiths, Language subscale
Can recognize everyday sounds	Not found anywhere else	

	Can locate him/herself in his/her immediate vicinity	Has difficulty finding his/her way, even in well-known places	5-15 Questionnaire, Domain Perception
	Knows the meaning of morning/evening<br>Knows the meaning of present/past/future	Has a diffuse idea of time, such as what time it is, whether it is morning or afternoon, whether it is time to go to school	5-15 Questionnaire, Domain Perception
	Can tell time mechanically	Can tell time mechanically (but does not understand the meaning of time)	5-15 Questionnaire, Domain Perception
	Is able to plan his/her time	Has uncertain perception of time, e.g. uncertain perception of how long 5 minutes or 1 hour is<br>Asks several times about when something is going happen	5-15 Questionnaire, Domain Perception
	Structures his/her day/time with the help of concrete objects, photographs, drawings, Bliss, written words	Not available anywhere else. The task is still of interest, as it can provide information and ideas for others	
PREREQUISITES FOR LEARNING			
Gatherings/group activities	Shows interest	Shows interest in novel objects or new people<br>Shows interest in the activities of others	Vineland, Domain Socialization

	Participates actively<br>Participates without disturbing	How does the child/pupil function in free activity, in controlled activity?	The Gotland material (Gotlandsmaterialet)
	Manages without an adult next to him/her	How does the child/pupil manage to work independently?	The Stockholm material (Stockholmsmaterialet), Independent work
	Can handle group size > 15 people, 5-15 people, <5 people	How does the pupil function in large groups, in small groups, one-to-one?	The Gotland material (Gotlandsmaterialet)
Individual work	Displays ability to concentrate	Often does not follow through on instructions and fails to finish schoolwork, chores, or duties	SNAP IV
		Failing to finish the activity started, can concentrate only for a short time	Conners scale
	Works independently	Is dependent<br>Dependent on constant confirmation, wants to know if he/she is doing right<br>Cannot take responsibility for his/her tasks, needs a lot of supervision	5-15 Questionnaire, Domain Executive functions
	Is able to start, execute and finish his/her tasks	Has difficulty planning the execution of an activity	5-15 Questionnaire, Domain Executive functions

		Has difficulty performing actions in several stages<br>Has difficulty getting started<br>Has difficulty completing activities	
	Is able to organize his/her work	Has difficulty organizing his/her work with tasks and activities<br>Has difficulty planning and organizing his/her learning, for example in what order the sub-tasks are to be done, what time is needed to complete the tasks	5-15 Questionnaire, Domain Executive functions
Ability to assimilate information	Understands and can use oral information	Has difficulty understanding explanations and instructions<br>Has a hard time understanding what someone said<br>Often hears wrong, misinterprets words	5-15 Questionnaire, Domain Language
	Remembers given information	Has difficulty remembering people's names,...historical facts,…long instructions	5-15 Questionnaire, Domain Memory
	Shows motivation	Is unmotivated for school work or similar learning situations	5-15 Questionnaire, Domain Learning
	Shows initiative	How is the student's initiative in learning situations?<br>How is the child /pupil's initiative?	The Stockholm material (Stockholmsmaterialet), Behaviour

		The Stockholm material (Stockholmsmaterialet), Social interaction with peers and adults
Shows attentiveness	Often has difficulty sustaining attention in tasks or play activities<br>Is often distracted by external stimuli<br>Often fails to pay close attention to details or makes careless mistakes in schoolwork or tasks	SNAP IV
Has trouble getting started	Has difficulty getting started with a task/activity	5-15 Questionnaire, Domain Executive functions
Needs repetition	Not found in other forms. This item is a complement to the item Remember given information	
Needs concrete material	Has difficulty solving abstract tasks (i.e. is dependent on concrete learning materials)<br>Has difficulty understanding or using abstract terms	5-15 Questionnaire, Domain Learning
Needs one-to-one tuition	Does the student need individual instruction?	The Stockholm material (Stockholmsmaterialet),

			Oral and written instruction individually and in group
	Understands and can use written information	How does the child/pupil handle oral/written instructions?	The Stockholm material (Stockholmsmaterialet), Oral and written instruction individually and in group
Reading ability	Can sound<br>Knows all sounds/phonemes	Linguistic awareness - letter-sound, phonological awareness	The Gotland material (Gotlandsmaterialet)<br>Language development
	Can read	Reads simple stories aloud	Vineland, Domain Communication
	Has reading comprehension	Has difficulty understanding what he / she is reading	5-15 Questionnaire, Domain Learning
	Likes to read	Does not enjoy reading<br>Reads on own initiative	5-15 Questionnaire, Domain Learning<br>Vineland, Domain Communication
	Reads slowly	Has difficulty reading at the right speed	5-15 Questionnaire, Domain Learning
	Guesses	Does a lot of guessing while reading/writing	5-15 Questionnaire, Domain Learning
Writing skills	Can spell	Has difficulty spelling	5-15 Questionnaire, Domain Learning

	Can form letters, write neatly	Has difficulty forming letters, writing neatly	5-15 Questionnaire, Domain Learning
	Can express him/herself in writing in an age-appropriate way		5-15 Questionnaire, Domain Learning
Mathematical skills	Knows addition<br>Knows subtraction<br>Knows multiplication<br>Knows division	The four arithmetic methods	The Gotland material (Gotlandsmaterialet)<br>Mathematical development
	Can handle problem solving	Problem solving<br>Difficulty using arithmetic rules<br>Difficulty handling given numbers	5-15 Questionnaire, Domain Learning<br>The Gotland material (Gotlandsmaterialet)<br>Mathematical development
	Has mathematical awareness	Mathematical awareness – counting using songs/rhymes, numerical perception, position system, calculation, observing patterns/ constancy of form	The Gotland material (Gotlandsmaterialet)<br>Mathematical development
	Understands and is able to use units	Units of measure	The Gotland material (Gotlandsmaterialet)<br>Mathematical development

Physical education	Has difficulty/does not like to participate in team sports such as football, rounders, floorball	Has difficulty / does not like to participate in team sports such as football, rounders, floorball	5-15 Questionnaire, Domain gross motor skills<br>The Gotland material (Gotlandsmaterialet)<br>Development in other subjects

Sources used for content validation

For the purpose of content validation several sources have been used. Some are tests and scales frequently used for investigation and assessments in e.g. child- and adolescent psychiatry/psychology, schools and habilitation clinics, most of them well known internationally.
In addition to this, some Swedish materials have also been used, e.g. support material for various pedagogical investigations, such as basic pedagogical assessments in school and extensive assessments prior to admission to special school. For some items, we have used a description in a Swedish bachelor degree project in educational science, Gothenburg University.
The content validation began in the beginning / middle of the 2010s. Some tests have since been revised. Due to this, the wording of some items might have been changed, but the gist is however the same. Sometimes domain names have also been changed.

Short description of the tests and materials used for content validation

Autism Spectrum Screening Questionnaire, (ASSQ - rev)
ASSQ was developed by Ehlers and Gillberg and further developed in collaboration with Lorna Wing in order to study the prevalence of Asperger's Syndrome. An extended version (ASSQ-REV) has been developed with the aim of identifying girls with autism at an earlier age. The questionnaire is developed for use with children and adolescents aged 4-18 yrs.

Available at:
https://www.gu.se/gnc/gncs-resurser/screeningformular/assq-autism-spectrum-screening-questionnaire
https://psykiatristod.se/download/18.26e8250017c6f95090f81ec/1634134959260/ASSQ.pdf (A-form)

Conners Parent Rating Scale (CPRS-R)

The form is used for the investigation of ADHD and provides information about the areas of inattention, hyperactivity / impulsivity, learning problems, executive functions, aggression and peer relationships. It can also be used for screening and follow-up. Available in a version containing 59 items (1), revised 1997 and also in a shorter version containing 28 items (2), also revised 1997, and finally there is one version with 10 items (3).

Available at:
http://www.pediatricenter.com/assets/forms/Conners_Parent_Rating.pdf (1)
https://www.stevensonwaplak.com/wp-content/uploads/2011/03/connorsteacher.pdf (2)
https://doi.org/10.1111/j.1651-2227.2008.01214.x (3)

The Griffith Mental Development Scales (GMDS)

The instrument consists of Griffith's development scale I, for children 0 to 2 years and Griffith's development scale II for children 2 to 7 years, and is a general developmental assessment for children up to 8 years. The assessment focuses on abilities and indicates ages when a child with age-typical development should have achieved a certain ability. The test contains five subscales; A Motor skills, B Personal and social behavior, C Hearing and speech, D Coordination of eye and hand, E Performance and scale II also F Practical reasoning. GMDS is used in assessment of preschool children in, for example, child health care and habilitation.

Available at:
https://www.hogrefe.com/uk/shop/griffiths-scales-of-child-development-third-edition.html

Orofacialt observationsschema (Orofacial observation form), Mun H center

Mun-H-center is a national center for oral health and orofacial function in rare diagnoses and is part of the public specialist dental care in Västra Götaland, Sweden. Mun-H-center collects data through examination forms, clinical observations and information from parents. The information stored in a database, which in 2021, contained data from more than 4,000 people with rare diagnoses.

Available at:
https://www.mun-h-center.se/en/research-and-facts/rare-diseases/

Pediatric Evaluation of Disability Inventory (PEDI)
PEDI is a standardized instrument with the aim of assessing the child's functional skills, degree of need for help in various activities as well as necessary aids and adaptations in the environment for the implementation of the activities. PEDI is intended for use with children aged 6 months to 7.5 years, but can also be used for the elderly. PEDI is used in habilitations and within schools.

Available at:
https://www.pearsonassessments.com/store/usassessments/en/Store/Professional-Assessments/Developmental-Early-Childhood/Pediatric-Evaluation-of-Disability-Inventory/p/100000505.html

Sense of Coherence Scale (SOC)
SOC is a form developed by Antonovsky that measures the degree of SOC. High values mean that the individual has a strong sense of coherence and thus a high ability to mangage challenges. The form contains 29 statements.

Reference:
Antonovsky A. (1987). Unraveling the Mystery of Health, Jossey-Bass Inc Publishers.

Swanson, Nolan and Pelham Teacher and Parent Rating Scale – IV (SNAP-IV)
The form is used in screening for ADHD and defiance syndrome. Here the version with 26 questions have been used. The questions are answered by parents and teachers and are intended for children 5 to 11 years. All items are taken from DSM-IV on attention deficit, impulsivity, defiance syndrome.

Available at:
http://www.shared-care.ca/files/Scoring_for_SNAP_IV_Guide_26-item.pdf

Vineland Adaptive Behavior Scales (VABS)
The VABS are intended for assessment of social/adaptive behavior in children and adults in investigations of intellectual disability. The scales contain an assessment of five behavioral aspects and assess abilities in communication, every day, social and motor skills as well as maladaptive behavior. The scales are used for people 2 to 21 years of age for investigation both in habilitation and in school.

Available at:
https://www.pearsonclinical.se/vineland-ii

5-15 Questionnaire for Evaluation of Development and Behaviour, Teachers' questionnaire
The form highlights development in motor skills, executive functions, perception, memory, language, social skills and learning, behavior and the occurrence of any emotional problems. It consists of statements, which express difficulties with a particular task or in a particular context. The form is used when assessing children, 5 to 15 years, the 5-15 form and 5 to 17 years, 5-15 R, with behavioral problems or developmental abnormalities, for example in psychological assessments in school.

Available at:
https://www.5-15.org/pdf/515_en-GB_teacher.pdf
https://www.5-15.org/pdf/manuals/515manual_en-GB.pdf

Basic pedagogical investigation Service material, Primary school (Pedagogisk basutredning Servicematerial, Grundskola)
The purpose is to provide an in-depth picture of the student's strengths and difficulties when a student deviates from age-typical development. The material contains areas of observations, such as pedagogical environment, language, interaction, group behavior, working methods (concentration, endurance, attention, instructions, start-end, group size, motor skills). Gotland Municipality, Children and Education Administration, Children and Student Health, January 2009.

No longer available in original digital form, please contact the main author for paper copy.

Available at:
https://docplayer.se/18308089-Pedagogisk-basutredning.html

Support material - pedagogical assessment prior to admission to a special primary school in the city of Stockholm (Stödmaterial - pedagogisk bedömning inför mottagande i grundsärskola i Stockholms stad)
In order for a student to be admitted to the special school, an interprofessional assessment is required, ie pedagogical, psychological, social and medical, according to the National Agency for Education's General Council. The pedagogical part aims to assess whether the student has the prerequisites to reach the knowledge requirements in compulsory school. It includes description and analysis of background, extra adaptations, special support, knowledge development, communication skills, social skills, judgment, behavior, independent work, ability to concentrate, everyday skills, sight, hearing and motor skills.

No longer available in digital form, please contact the main author for paper copy.
A revised version of the material is available at:
https://leverantor.stockholm/globalassets/foretag-och-organisationer/leverantor-och-utforare/verksamhetsomraden/fristaende-skola/grundskola/grundsarskola/utbf8655-stodmaterial-pedagogisk-bedomning.pdf

Autism and communication - a qualitative interview study with 7 educators in preschool and special school (Autism och kommunikation – en kvalitativ intervjustudie med 7 pedagoger inom förskolan och särskolan)
The work describes, among other things, the language development of children with autism as well as different ways of communicating with and means for AAC.

Bang L, Boij V. (2006). Autism och kommunikation (Degree project, bachelor degree in Educational science), Gothenburg University

Available at:
https://gupea.ub.gu.se/bitstream/2077/3843/1/HT06-2611-064.pdf
