# Supplementary material for: Consequences of rare diagnoses for education and daily life: development of an observation instrument
Source: Orphanet J Rare Dis. 2022 Apr 12;17:165. doi: 10.1186/s13023-022-02303-y (PMC9004121; doi:10.1186/s13023-022-02303-y)
Supplement: Supplementary file 4 — Additional file 4. Descriptive statistics. [file 13023_2022_2303_MOESM4_ESM.pdf]

Additional file 4. Descriptive statistics. Frequencies (*n*), means (*m*), and standard deviations (*sd*) for the eleven domains/subdomains subdivided by diagnosis and gender.

Diagnosis	Social and communication ability		Emotion and behaviours		Communication and language		Ability to manage his/her disability		Activities of Daily Life		Gross motor skills		Fine motor skills		Perception and worldview		Gatherings/ Group activities		Individual work		Ability to assimilate information	
	n	***m (sd)***	***n***	***m (sd)***	***n***	***m (sd)***	***n***	***m (sd)***	***n***	***m (sd)***	***n***	***m (sd)***	***n***	***m (sd)***	***n***	***m (sd)***	***n***	***m (sd)***	***n***	***m (sd)***	***n***	***m (sd)***
**Achondro**	20	1.13 (0.27)	20	1.24 (0.32)	20	1.25 (0.44)	19	1.23 (0.35)	19	1.25 (0.62)	20	1.65 (0.38)	20	0.85 (0.81)	20	1.01 (0.03)	20	1.09 (0.22)	20	1.41 (0.75)	20	1.57 (0.52)
Male	12	1.21 (0.33)	12	1.02 (0.40)	12	1.25 (0.45)	12	1.31 (0.42)	11	1.34 (0.76)	12	1.66 (0.45)	12	0.75 (0.75)	12	1.01 (0.03)	12	1.14 (0.28)	12	1.58 (0.91)	12	1.55 (0.56)
Female	8	1.00 (0.00)	8	1.15 (0.04)	8	1.25 (0.46)	7	1.10 (0.13)	8	1.13 (0.35)	8	1.62 (0.29)	8	1.00 (0.93)	8	1.01 (0.03)	8	1.02 (0.05)	8	1.16 (0.35)	8	1.59 (0.49)
**EDS**	32	1.47 (0.49)	32	1.52 (0.53)	32	1.38 (0.55)	32	1.71 (0.55)	31	1.11 (0.24)	32	1.49 (0.42)	32	0.59 (1.01)	31	1.18 (0.34)	32	1.59 (0.69)	32	1.84 (0.82)	32	1.69 (0.56)
Male	23	1.58 (0.50)	23	1.65 (0.53)	23	1.48 (0.59)	23	1.81 (0.57)	22	1.14 (0.28)	23	1.48 (0.46)	23	0.65 (1.15)	22	1.23 (0.38)	23	1.71 (0.69)	23	2.10 (0.81)	23	1.81 (0.53)
Female	9	1.17 (0.34)	9	1.19 (0.39)	9	1.11 (0.33)	9	1.43 (0.41)	9	1.03 (0.08)	9	1.53 (0.30)	9	0.44 (0.53)	9	1.07 (0.20)	9	1.30 (0.65)	9	1.19 (0.33)	9	1.37 (0.54)
**FX**	44	2.90 (0.71)	44	2.37 (0.64)	44	3.18 (0.87)	36	2.62 (0.62)	44	2.03 (0.97)	44	1.62 (0.64)	44	1.41 (1.19)	44	1.96 (0.74)	44	3.02 (0.73)	44	3.41 (0.74)	44	3.16 (0.56)
Male	34	3.03 (0.62)	34	2.51 (0.57)	34	3.38 (0.78)	27	2.75 (0.46)	34	2.20 (0.94)	34	1.70 (0.63)	34	1.65 (1.23)	34	2.10 (0.68)	34	3.25 (0.49)	34	3.67 (0.40)	34	3.31 (0.40)
Female	10	2.46 (0.82)	10	1.90 (0.68)	10	2.50 (0.85)	9	2.24 (0.87)	10	1.46 (0.87)	10	1.35 (0.64)	10	0.60 (0.52)	10	1.48 (0.78)	10	2.24 (0.89)	10	2.53 (0.96)	10	2.64 (0.71)
**Narco**	72	1.29 (0.37)	72	1.41 (0.49)	72	1.13 (0.33)	69	1.72 (0.49)	72	1.03 (0.08)	71	1.17 (0.23)	72	0.03 (0.17)	71	1.05 (0.10)	72	1.40 (0.44)	72	1.81 (0.61)	72	1.67 (0.48)
Male	42	1.32 (0.41)	42	1.42 (0.53)	42	1.14 (0.35)	40	1.75 (0.53)	42	1.02 (0.07)	42	1.13 (0.20)	42	0.02 (0.15)	41	1.04 (0.08)	42	1.47 (0.51)	42	1.93 (0.59)	42	1.73 (0.48)
Female	30	1.24 (0.29)	30	1.39 (0.43)	30	1.10 (0.31)	29	1.67 (0.45)	30	1.05 (0.10)	30	1.22 (0.27)	30	0.03 (0.18)	29	1.07 (0.13)	30	1.31 (0.31)	30	1.64 (0.60)	30	1.59 (0.47)
**NF**	12	2.06 (0.65)	12	1.87 (0.77)	11	2.09 (0.83)	12	2.13 (0.62)	12	1.33 (0.54)	12	1.62 (0.47)	12	0.42 (0.90)	12	1.63 (0.62)	12	2.20 (0.87)	11	2.27 (0.86)	12	2.34 (0.82)
Male	5	2.09 (0.62)	5	2.03 (0.88)	4	2.25 (0.96)	5	1.97 (0.59)	5	1.35 (0.38)	5	1.65 (0.46)	5	0.20 (0.45)	5	1.70 (0.54)	5	2.17 (0.89)	5	2.40 (0.89)	5	2.28 (1.00)
Female	7	2.03 (0.72)	7	1.77 (0.73)	7	2.00 (0.82)	7	2.24 (0.66)	7	1.32 (0.66)	7	1.59 (0.51)	7	0.57 (1.13)	7	1.58 (0.71)	7	2.22 (0.93)	6	2.17 (0.90)	7	2.37 (0.76)
**Noonan**	11	2.50 (0.65)	11	2.22 (0.85)	11	2.27 (0.90)	11	2.55 (0.59)	11	1.75 (0.77)	11	1.71 (0.57)	11	0.91 (1.22)	11	1.88 (0.58)	11	2.56 (0.66)	11	2.89 (0.72)	11	2.72 (0.62)
Male	3	2.48 (1.13)	3	1.63 (0.13)	3	2.33 (1.53)	3	2.34 (0.75)	3	1.58 (1.01)	3	1.92 (0.96)	3	1.00 (1.73)	3	2.09 (0.90)	3	2.57 (1.03)	3	2.83 (0.88)	3	2.73 (0.93)
Female	8	2.51 (0.48)	8	2.45 (0.90)	8	2.25 (0.71)	8	2.63 (0.55)	8	1.81 (0.73)	8	1.64 (0.41)	8	0.88 (1.13)	8	1.80 (0.47)	8	2.55 (0.57)	8	2.91 (0.72)	8	2.71 (0.55)
**PWS**	22	2.40 (0.63)	22	2.18 (0.74)	22	2.77 (0.92)	14	2.51 (0.42)	22	2.31 (1.01)	22	2.01 (0.61)	22	1.68 (1.49)	22	1.95 (0.54)	22	2.55 (0.83)	22	2.85 (0.76)	22	2.91 (0.63)
Male	7	2.70 (0.65)	7	2.75 (0.70)	7	3.14 (0.90)	5	2.80 (0.46)	7	3.14 (0.84)	7	2.23 (0.80)	7	2.57 (1.51)	7	2.21 (0.53)	7	2.98 (0.57)	7	3.39 (0.50)	7	3.39 (0.30)
Female	15	2.27 (0.59)	15	1.91 (0.61)	15	2.60 (0.91)	9	2.35 (0.32)	15	1.92 (0.85)	15	1.90 (0.50)	15	1.27 (1.33)	15	1.82 (0.52)	15	2.35 (0.87)	15	2.60 (0.74)	15	2.69 (0.62)
**Williams**	43	2.61 (0.52)	43	2.44 (0.60)	43	2.42 (0.82)	39	2.41 (0.43)	43	2.09 (0.90)	43	1.81 (0.57)	43	1.67 (1.41)	43	2.11 (0.50)	43	2.98 (0.57)	43	3.25 (0.51)	43	3.15 (0.43)
Male	15	2.77 (0.41)	15	2.70 (0.54)	15	2.53 (0.74)	13	2.44 (0.49)	15	2.13 (0.99)	15	2.00 (0.53)	15	2.47 (1.41)	15	2.21 (0.50)	15	3.08 (0.35)	15	3.33 (0.39)	15	3.30 (0.29)
Female	28	2.53 (0.56)	28	2.31 (0.59)	28	2.36 (0.87)	26	2.40 (0.41)	28	2.07 (0.86)	28	1.70 (0.58)	28	1.25 (1.24)	28	2.05 (0.49)	28	2.92 (0.66)	28	3.21 (0.57)	28	3.07 (0.48)
**22q11**	11	2.12 (0.65)	11	1.97 (0.48)	11	3.00 (0.63)	11	2.38 (0.40)	11	1.61 (0.91)	11	1.32 (0.46)	11	0.82 (1.08)	11	1.77 (0.53)	11	2.16 (0.68)	11	2.80 (0.53)	11	2.57 (0.60)
Male	6	2.18 (0.82)	6	1.71 (0.48)	6	3.33 (0.52)	6	2.17 (0.32)	6	1.79 (1.19)	6	1.38 (0.59)	6	1.17 (1.17)	6	1.79 (0.67)	6	2.05 (0.89)	6	2.71 (0.62)	6	2.52 (0.73)
Female	5	2.04 (0.46)	5	2.28 (0.29)	5	2.60 (0.55)	5	2.63 (0.36)	5	1.40 (0.45)	5	1.24 (0.28)	5	0.40 (0.89)	5	1.75 (0.37)	5	2.29 (0.34)	5	2.90 (0.45)	5	2.64 (0.48)
**Total**	267	1.99 (0.85)	267	1.87 (0.74)	266	2.02 (1.05)	243	2.06 (0.67)	265	1.57 (0.84)	266	1.54 (0.54)	267	0.87 (1.20)	265	1.55 (0.64)	267	2.13 (0.95)	266	2.47 (0.99)	267	2.36 (0.87)
Male	147	2.04 (0.90)	147	1.92 (0.77)	146	2.11 (1.14)	134	2.07 (0.68)	145	1.61 (0.90)	147	1.55 (0.58)	147	1.00 (1.31)	145	1.57 (0.68)	147	2.20 (0.97)	147	2.61 (0.99)	147	2.41 (0.90)
Female	120	1.91 (0.78)	120	1.80 (0.71)	120	1.90 (0.92)	109	2.04 (0.66)	120	1.52 (0.75)	119	1.53 (0.50)	120	0.72 (1.03)	120	1.53 (0.59)	120	2.05 (0.91)	119	2.30 (0.96)	120	2.31 (0.83)

Notes. Abbreviations: Achondro = Achondroplasia; EDS = Ehlers Danlos syndrome; FX = Fragile X-syndrome; Narco = Narcolepsy; NF = Neuro-fibromatosis type 1; Noonan = Noonan syndrome; PWS = Prader Willi syndrome; Williams = Williams syndrome; 22q11 = 22q11 deletion syndrome;
