# Supplementary material for: Consequences of rare diagnoses for education and daily life: development of an observation instrument
Source: Orphanet J Rare Dis. 2022 Apr 12;17:165. doi: 10.1186/s13023-022-02303-y (PMC9004121; doi:10.1186/s13023-022-02303-y)
Supplement: Supplementary file 5 — Additional file 5. Interrater reliability. [file 13023_2022_2303_MOESM5_ESM.pdf]

Additional file 5. Interrater reliability

		male, 16 yrs	male, 16 yrs	male, 15 yrs	female, 12 yrs	male, 10 yrs	male, 8 yrs	
**Domain**	**number of items**	**equally rated items**	**equally rated items**	**equally rated items**	**equally rated items**	**equally rated items**	**equally rated items**	**% equally rated items**
Social/communicative ability	10	7	9	9	9	8	10	86,7%
Emotions and behaviours	9	7	9	8	9	9	9	94,4%
Communication and language	8	7	7	8	8	8	8	95,8%
Ability to manage disability and everyday life	9	7	7	9	7	7	8	83,3%
Activities of daily life, ADL	4	4	3	4	4	4	2	87,5%
Gross motor skills	13	12	10	10	10	13	13	87,2%
Fine motor skills	6	6	6	6	6	4	6	94,4%
Perception and worldview	19	17	15	17	18	19	17	90,4%
Prerequisites for learning, tot	22	22	19	22	22	19	22	95,5%
Total	**100**	**89**	**85**	**93**	**93**	**91**	**95**	**91,0%**
