# Supplementary material for: Consequences of rare diagnoses for education and daily life: development of an observation instrument
Source: Orphanet J Rare Dis. 2022 Apr 12;17:165. doi: 10.1186/s13023-022-02303-y (PMC9004121; doi:10.1186/s13023-022-02303-y)
Supplement: Supplementary file 6 — Additional file 6. Multinomial regression analysis. [file 13023_2022_2303_MOESM6_ESM.pdf]

Additional file 6. Multinomial regression with narcolepsy as the reference group and the eleven domains/subdomains as independent variables.

	Domain/subdomain (IVs)											
	Intercept	Social and communic-ation ability	Emotions and behaviours	Communic-ation and language	Ability to manage his/her disability	Activities of Daily Life	Gross motor skills	Fine motor skills	Perception and worldview	Gatherings / Group activities	Individual work	Ability to assimilate informa-tion
Diagnosis(DVs)												
Achondro	12.317	-1.100	5.343*	1.869	-7.411**	2.877	4.053*	4.886***	-20.615	-2.293	-1.363	3.266†
	(12.458)	(2.449)	(2.561)	(1.323)	(2.627)	(2.341)	(1.702)	(1.303)	(12.577)	(2.326)	(1.298)	(1.753)
EDS	-5.322*	-0.116	1.127	1.054†	-2.048*	-1.380	2.863**	3.244***	1.466	1.063	-0.794	0.321
	(2.183)	(0.950)	(1.104)	(0.638)	(0.984)	(1.785)	(1.047)	(0.972)	(1.946)	(0.919)	(0.750)	(0.984)
FX	-16.710***	3.952***	-1.297	2.651***	-2.357†	0.156	-2.193	2.234*	5.368**	0.711	-0.360	2.315†
	(3.952)	(1.234)	(1.251)	(0.782)	(1.309)	(1.635)	(1.540)	(1.044)	(2.087)	(1.192)	(1.071)	(1.250)
NF	-10.990***	1.168	-0.558	1.241	-1.198	0.164	-0.193	2.000†	5.968**	1.693	-2.606*	1.874
	(2.556)	(1.223)	(1.345)	(0.781)	(1.338)	(1.741)	(1.525)	(1.069)	(2.135)	(1.232)	(1.121)	(1.285)
Noonan	-15.138***	2.928*	-1.639	0.454	-0.171	0.294	0.019	2.340*	5.163*	0.876	-0.740	1.700
	(3.015)	(1.320)	(1.341)	(0.834)	(1.430)	(1.643)	(1.562)	(1.045)	(2.128)	(1.278)	(1.164)	(1.314)
PWS	-15.577***	2.569†	-1.047	1.823*	-2.112	0.617	0.946	2.292*	4.193†	0.925	-2.115†	3.473*
	(3.053)	(1.326)	(1.326)	(0.824)	(1.422)	(1.651)	(1.558)	(1.059)	(2.200)	(1.273)	(1.156)	(1.425)
Williams	-14.182***	2.221*	-0.555	0.253	-2.993*	0.444	-0.409	2.800**	5.591**	1.998†	-1.677†	3.689**
	(2.788)	(1.122)	(1.200)	(0.743)	(1.251)	(1.626)	(1.440)	(1.025)	(2.023)	(1.080)	(0.966)	(1.235)
22q11	-14.066***	2.219	-1.410	3.640***	-1.138	0.280	-4.128*	1.846†	8.333***	-0.742	-0.095	0.580
	(3.328)	(1.439)	(1.617)	(0.992)	(1.564)	(1.784)	(1.899)	(1.115)	(2.406)	(1.573)	(1.380)	(1.600)
LR tests												
-2 LL	560.70	476.34	469.49	503.02	485.60	467.93	491.62	495.54	493.67	469.46	471.21	477.61
Chi-square (Df = 8)	100.89***	16.53*	9.68	43.21***	25.79***	8.12	31.86***	35.73***	33.86***	9.65	11.40	17.80*
Model fit info												
-2 LL (intercept)	940.66											
-2 LL (final model)	459.81											
χ^2 (Df)	480.85*** (88)											
Nk. R^2	0.89											
Dev. χ^2(Df)	459.81 (1776)											
P. χ^2 (Df)	3290.28*** (1776)											
N	237											

Notes. ***p≤.001, **p ≤.01, *p≤.05, †p≤.10. Regression coefficients (logged odds) with associated standard errors in parenthesis are presented in the table. Narcolepsy is used as the reference group in the analysis. Abbreviations: Achondro = Achondroplasia; ED = Ehlers Danlos syndrome; FX = Fragile X-syndrome; NF = Neurofibromatosis type 1; Noonan = Noonan

syndrome; PW = Prader Willi syndrome; 22q11 = 22q11 deletion syndrome; Williams syndrome; LR = Liklihood ratio; LL = Log likelihood; Nk = Nagelkirk; P = Pearson; Dev = Deviance; Df = Degrees of freedom.
