# Supplementary material for: Consequences of rare diagnoses for education and daily life: development of an observation instrument
Source: Orphanet J Rare Dis. 2022 Apr 12;17:165. doi: 10.1186/s13023-022-02303-y (PMC9004121; doi:10.1186/s13023-022-02303-y)
Supplement: Supplementary file 7 — Additional file 7. Summary of contributions of the domains. [file 13023_2022_2303_MOESM7_ESM.pdf]

Additional file 7. Summary of significant (<0.05) contributions of the different domains/subdomains to each of the eight included diagnoses[1]

	Domain/subdomain										
**Diagnosis**	Social and communicative ability	Emotions and behaviours	Communication and langauge	Ability to manage his / her disability and everyday life	Activity of Daily Life	Gross motor skills	Fine motor skills	Perception and worldview	Gatherings/ Group activity	Individual work	Ability to assimilate information
Achondroplasia		X		X		X	X				
Ehlers Danlos Syndrome				X		X	X				
Fragile X syndrome	X		X				X	X			
Neurofibromatosis type 1								X		X	
Noonan syndrome	X						X				
Prader Willi syndrome			X				X				X
Williams syndrome	X			X			X	X			X
22q11 deletion syndrome			X			X		X			

[1]The reference category is Narcolepsy
